# How can a measure improve assessment and management of symptoms and concerns for people with dementia in care homes? A mixed-methods feasibility and process evaluation of IPOS-Dem

**DOI:** 10.1371/journal.pone.0200240

**Published:** 2018-07-11

**Authors:** Clare Ellis-Smith, Irene J. Higginson, Barbara A. Daveson, Lesley A. Henson, Catherine J. Evans, On behalf of BuildCARE

**Affiliations:** 1 Department of Palliative Care, Policy and Rehabilitation, Cicely Saunders Institute, King’s College London, London, United Kingdom; 2 King’s College Hospital NHS Foundation Trust, London, United Kingdom; 3 Sussex Community NHS Foundation Trust, Brighton, Sussex, United Kingdom; University of Mississippi, UNITED STATES

## Abstract

**Background:**

Assessment of people with dementia is challenging; with undetected and under treated symptoms and concerns resulting in avoidable distress, and few evidence-based interventions to support this. We aimed to understand the mechanisms of action of a measure to support comprehensive assessment of people with dementia in care homes; and its acceptability, feasibility, and implementation requirements.

**Methods:**

A qualitative study with an embedded quantitative component in three residential care homes, underpinned by an initial theoretical model of mechanisms of action. The measure, the Integrated Palliative care Outcome Scale for Dementia (IPOS-Dem), was introduced into the care of residents with dementia for 12 weeks. Qualitative data comprised focus groups and semi-structured interviews with family, care home staff, general practitioners and district nurses; and non-participant observations. Quantitative data comprised IPOS-Dem data. Directed content analysis for qualitative data, and descriptive statistics were used for quantitative data.

**Findings:**

Key mechanisms of action were: improved observation and awareness of residents, collaborative assessment, comprehensive ‘picture of the person’, systematic record keeping, improved review and monitoring, care planning and changes to care provision, and facilitated multi-agency communication. Potential benefit included improved symptom management, improved comprehensive care, and increased family empowerment and engagement. IPOS-Dem was found to be acceptable and feasible. It was perceived as quick and easy to use, with proportion of overall missing data decreasing from 2.1% to 1.1% from baseline to final time points. ‘Trust’ in the measure was important; and leadership essential to ensure integration into care processes.

**Conclusions:**

In a population with complex care needs, with challenges to assessment and barriers to multi-agency working, a measure introduced into routine care is feasible and acceptable, and supports assessment and management of symptoms and concerns. A refined theoretical model demonstrating the likely mechanisms of action was developed. Further evaluation is required to test its effectiveness.

## Introduction

Dementia is a progressive and terminal illness [1]. It is characterised by increasing dependence and disability [2, 3] meaning that 24-hour care is frequently required [4]. Worldwide demographic change of increasingly older population profiles will result in growing prevalence of dementia [5, 6]; with consequent increasing demand in care home provision, and increasing requirement for palliative care from non-specialist providers [6], including care home staff.

People with dementia may experience high symptom burden [7, 8] due to dementia, multi-morbidities [9] and side-effects of treatments. It is challenging to assess symptoms and concerns in people who are verbally compromised. This impedes practitioners’ ability to assess symptoms and concerns and often leads to under detection and treatment; and increased distress, behavioural changes and reduced quality of life [10]. Practice guidelines recommend comprehensive assessment and non-pharmacological interventions to identify and treat the underlying causes of behavioural changes such as agitation [11]. However there are few high-quality evidence-based interventions for care homes to improve comprehensive assessment and management of symptoms and concerns in this population [12–15]. Furthermore, in the UK particularly, there are barriers to accessing health care for residents in care homes that have no onsite nursing care and therefore rely on external providers, including General Practitioners/Physicians (GPs) and community/district nurses (DNs), with inconsistencies in provision across the country and challenges to integrated working [16].

Measures used in routine clinical care can facilitate assessment and change care processes leading to improved patient outcomes [17]. However, little is known of their use in care home settings [17]. Measures used in this way are complex interventions [18, 19]. In developing and evaluating complex interventions it is important to understand the likely mechanisms of action, and potential harms and safety of the intervention [19, 20], how the intervention should be implemented, and any influencing contextual factors [21, 22]. This is particularly the case in the care home sector where there are additional challenges of multi-agency, integrated working (including family members) [16, 23].

We aimed to explore the mechanisms of action, feasibility, acceptability and implementation requirements of a measure, the Integrated Palliative care Outcome Scale (IPOS-Dem), used in routine care to support comprehensive assessment of symptoms and concerns of care home residents with dementia and their family members.

## Methods

### Study design

**Fig 1:**
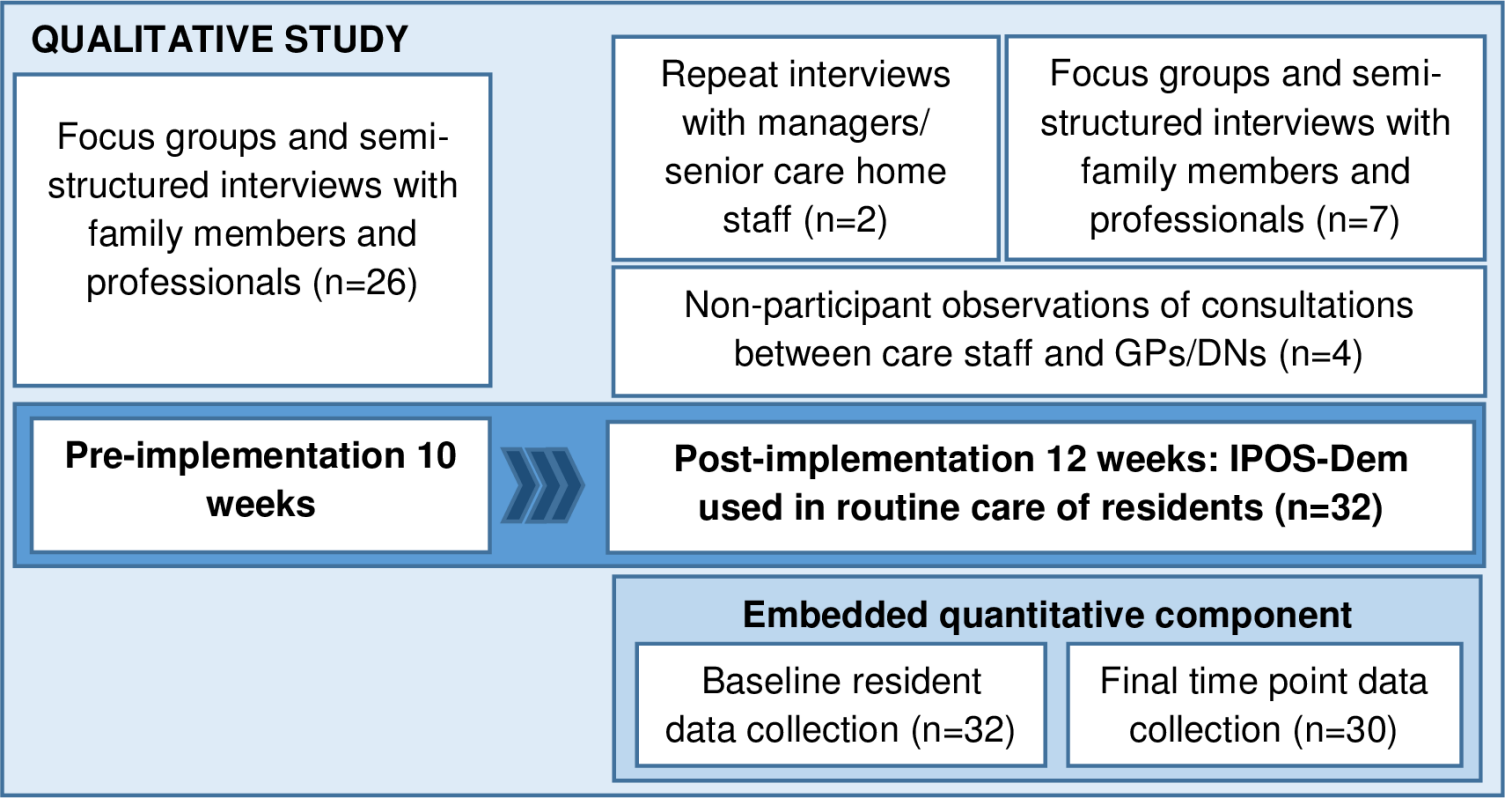
Study design and data collection.

We conducted a qualitative study with a concurrent embedded quantitative component [24] (Fig 1), underpinned by a theoretical model of the likely mechanisms of action of a measure used in routine care for people with dementia in care homes. The theoretical model was developed prior to the study commencing, derived from two theoretical models that proposed the likely mechanisms of action and outcomes of using a measure as part of routine care in other health care settings [25–27]. The theoretical model uses Theory of Change–a ‘theory of how and why an initiative works’[28] which presents a hypothetical causal pathway of expected mechanisms of action and intended outcomes presented in diagrammatic format. Theory of Change provides a framework for unpacking the ‘black box’ of a complex intervention [29], developed from research evidence or from stakeholder consultation [30].

IPOS-Dem was implemented into routine care of residents with dementia for 12 weeks. Qualitative data collection included pre-implementation focus groups and semi-structured interviews with families and professionals (care home staff, GPs, DNs); and post-implementation (during and towards the end of implementation) focus groups and semi-structured interviews with families and professionals, and non-participant observations. Quantitative data collection comprised measures with residents at baseline and at 12 weeks. Throughout the planning, data collection and analysis of the study, we consulted with experts including service users and carers, and academics and clinicians in palliative care, primary care and mental health.

### Setting

Three residential care homes registered to provide care for people aged 65 and over in a London borough, United Kingdom. Unlike nursing homes, residential care settings are not required to employ professionally qualified nurses, and therefore have no onsite nursing. The main health care providers are GPs and DNs [31]. Settings were recruited to obtain a variety of funding types, ownership and dementia-registration [32].

### Participant recruitment

Recruitment of residents was informed by the Mental Capacity Act [33]. Eligible residents were identified by senior staff in the care homes. The care home staff introduced residents to the research team. The research team met with residents to ascertain willingness to participate and assess mental capacity to consent for themselves. Those residents that had capacity gave written informed consent. As we anticipated that the majority of residents would only be able to consent in the moment, we took the approach of gaining the advice of consultees in addition to informed consent [34]. The care home therefore sent a letter on behalf of the research team to a close friend or family member to invite them to advise on whether the resident should participate in research (personal consultee). Two letters were sent. If no response was received after one week of the second letter being sent, a nominated consultee was asked to advise on resident participation [33]. The nominated consultee was independent from the research study and used all available information (including meeting with the resident, reviewing case notes and speaking to care home staff) in order to give advice on resident participation.

At each recruitment phase we advertised and held coffee mornings in the care homes to share study information with family (including friends) of residents with dementia (CES/CP) [35]. Interested family members shared their contact details with the research team. Post-implementation, we contacted family members acting as personal consultees in the recruitment of residents lacking capacity [33] who had expressed interest in participating and shared their contact details. We made up to two attempts to contact family members to recruit them. Recruitment of care home staff for both phases involved formal and informal meetings with managers and care home staff. GPs and DNs responsible for health care provision for the participating care settings were identified and invited to participate. All participants were provided with study information sheets at least 24 hours prior to participating, and gave written informed consent (CES/CP/LAH).

### Participant eligibility criteria

Participants for the focus groups and semi-structured interviews comprised family and professionals. Non-participant observations were conducted of meetings between GPs and/or DNs, and senior care home staff which were held to discuss and review residents’ care.

Managers or senior care home staff participated in repeat semi-structured interviews about feasibility and implementation requirements of using the measure.

All care home staff working at the participating settings were eligible to participate. Eligibility criteria for family were 18 years or older, able to provide consent and English-speaking. Additionally, post-implementation, family members were relatives or friends of residents recruited to receive IPOS-Dem intervention. Care home staff were purposively sampled to provide a range of seniority and care roles. GPs and DNs were eligible to participate if they were responsible for providing health care to the participating settings. All family and health professionals (GPs and DNs) meeting the inclusion criteria were invited to participate.

Eligibility criteria of residents were a permanent resident of the care home, formal diagnosis of dementia or cognitive impairment of stages four to seven on Functional Assessment Staging (FAST) [2].

### IPOS-Dem intervention

IPOS-Dem [27] formed the intervention. IPOS-Dem is a brief but comprehensive measure for use in routine care by care home staff without a nursing qualification (unqualified care home staff). Multiple assessments exist to assess individual symptoms including pain [36]. However there is a gap in comprehensive assessment to improve assessment and management of symptoms and concerns for people with dementia and multi-morbidities [14]. IPOS-Dem is developed from the Integrated Palliative care Outcome Scale (IPOS), a measure developed and used in clinical practice to inform the care of people with palliative care needs [37]. Rather than add to the plethora of measures used in palliative care populations [38] and dementia populations [14], IPOS-Dem adapts IPOS as the latest version of the POS family of comprehensive measures. These measures are established and validated and widely used in clinical practice and research nationally and internationally [39–41]. The adaptation includes dementia-specific symptoms and concerns (such as delusions) and symptoms common in older people with multi-morbidities (such as pain or constipation), and by unqualified care home staff who provide the majority of direct care in care homes. IPOS-Dem was developed through literature review, qualitative interviews and focus groups with stakeholders, and cognitive interviews with care home staff [27]. It comprises twelve questions covering common symptoms and concerns experienced by people with dementia over the past week. Each item is rated on a five-point scale from 0 (no problem) to 4 (very severe). The first question is unscored and is a free text response option of the main problems experienced by the resident. IPOS-Dem can be accessed and downloaded for free [42]. Our pre-implementation work examined an early version of IPOS-Dem [27] in terms of the likely mechanisms of action, and implementation requirements for routine care, and development of an instruction manual [27, 43]. The findings formed the final version explored in the implementation phase.

In the implementation phase, care home staff administered the final version of IPOS-Dem to all participating residents at baseline and final time point data collection at 12 weeks. Participating care home staff were aware that the measure was newly developed and under preliminary evaluation. During the implementation period, participating care homes were given IPOS-Dem and asked to use it, according to the instruction manual with recruited residents. The instruction manual recommends that IPOS-Dem is used monthly at the time of care plans, or flexibly at times of resident change. Apart from at the baseline and final time points, the research team was not involved and did not prompt care home staffs’ use of IPOS-Dem through the course of the implementation phase. This was to understand the implementation of IPOS-Dem without the use of facilitation [44] which is frequently not available or sustainable in under-resourced care settings [45].

### Demographic and clinical data collection

Family and professional demographic data:

Demographic data on family and professional participants were collected using standardised data collection forms, collected by the research team from participants. Demographic data for family included relationship to resident, gender and age. Professional demographic data were profession or role, years of experience, gender, and ethnicity.

Resident demographic and clinical data:

We used a data collection form and extracted demographic and clinical data from case notes at baseline. Demographic and clinical data included resident age, diagnosis of dementia (yes/no), type of dementia, gender, ethnicity, FAST dementia staging [2] (from case notes and care home staff), morbidities and medication.

### Qualitative data collection

Pre- and post-implementation focus groups and semi-structured interviews:

Separate focus groups were conducted with family and professionals. Focus groups were chosen as a method of data collection to obtain participant interaction data [46]. Semi-structured interviews were conducted to enhance data richness by alternative data collection methods [47]. The pre-implementation topic guide consisted of a PowerPoint [48] presentation on the purpose of IPOS-Dem. To stimulate discussion about how IPOS-Dem could benefit residents and family, and requirements for its properties and implementation, we used case vignettes within the topic guides (S1 File) [49]. Post-implementation topic guides were informed by the findings of the previous phase and also included questions on IPOS-Dem mechanisms of action, measurement properties and implementation requirements. Topic guides for the manager interviews were similar, but included additional questions on implementation requirements and feasibility within the care home setting and the resources available. All topic guides were reviewed by expert members, and piloted and refined before being used in the main study. Given the potentially sensitive nature of the focus groups and interviews, a distress protocol was developed. Focus groups and interviews were digitally recorded. All focus groups and the majority of semi-structured interviews were conducted in the care homes. One interview with a family member was conducted in her own home in accordance with her preference.

Non-participant observations:

Non-participant observations were conducted in the care homes of care home staff and health professionals discussing and reviewing residents to further understand the process of integrated professional working, and a means of data triangulation. Observations of consultations between senior care home staff and visiting health professionals occurred at regular intervals through the intervention period and field notes made.

All qualitative data collection was conducted by CES (female; BSc, MSc), an Occupational Therapist with clinical experience in older adult mental health and dementia, who at the time of the study was a PhD Training Fellow. A second researcher (CP/LAH) was present for focus groups to record observations and implement the distress protocol if required. Data collection continued until data saturation was achieved. This was defined as the point where no new themes or subthemes were being generated from further data collection [50, 51].

### Quantitative data collection

Data collection time points (Fig 1):

Resident baseline data collection occurred just prior to implementation of IPOS-Dem in routine care. Final time point data collection occurred at 12 weeks at the end of implementation of IPOS-Dem. Baseline data collection consisted of a measure of agitation, the Cohen-Mansfield Agitation Inventory [52] and a measure of function, the Barthel Index [53]. At both time points, IPOS-Dem with an attached utility questionnaire, were completed by care home staff independently from the research team.

IPOS-Dem utility questionnaire:

For the purposes of the study, a brief utility questionnaire was included at the end of each IPOS-Dem to be completed by care home staff using the measure. This comprised four brief questions regarding the acceptability and usefulness of IPOS-Dem (S2 File).

### Qualitative data analysis

All recordings were transcribed verbatim and detailed field notes were made, and entered into Nvivo 10 [54] to aid data management and analysis. Transcripts were checked against the original recordings by CES to ensure accuracy. Data were analysed using directed content analysis [55]. A coding framework was developed informed by the theoretical model [27]. Additional codes were developed during analysis for relevant data that could not be coded into the existing framework [55]. One researcher conducted all coding (CES). A second researcher (CJE) coded a selection of interviews independently. Where coding differed, this was discussed and reviewed until consensus was reached and the coding framework was revised based on consensus. The full set of transcripts were then recoded using the finalised coding framework (CES). The codes were then inductively categorised into themes and subthemes. To triangulate the data, family and professional data, and pre-implementation and post-implementation data were compared and contrasted [56]. All analysis was discussed in regularly held research supervision meetings (CJE, BAD, IJH) to enhance reflexivity and ensure accurate representation of findings.

### Quantitative data analysis

Quantitative data were analysed using simple descriptive statistics for demographic, clinical, IPOS-Dem and IPOS-Dem utility questionnaire data. To explore for patterns of missing baseline IPOS-Dem data [57] (all missing, ‘missing—cannot assess’, ‘missing–reason unknown’) across cases, we used *X*^*2*^ or Fisher’s exact test with a Bonferroni-corrected alpha level of p<0.003. Assumptions (normality, outliers) were tested prior to all analysis. Pearson’s r and paired t-test (for parametric) or Spearman’s rho and Wilcoxon signed ranked test (for non-parametric data) were used to examine correlation and mean difference between baseline and final time points scores respectively with Bonferroni-corrected alpha level of p<0.004. Analyses were conducted both without imputation and using two methods of imputation (mean item score and mean case score) [57]. All analyses were conducted using SPSS version 22 [58].

### Ethics

Ethical approval was obtained from the National Research Ethics Committee–London South East, a committee flagged for adults lacking capacity [NRES: 13/LO/1339], and Local Authority Research Governance Framework approval was obtained for research in social care settings. Where required, individual care home setting ethical approval was obtained. National Health Service (NHS) research governance approval was obtained for participating NHS staff from the respective NHS employers.

## Findings

### Settings

Recruited care home size ranged from 26–33 beds. No relationship existed between the research team and care homes prior to the study commencing. Pre-implementation data collection commenced in May 2014 and completed in July 2014. Resident recruitment commenced in July 2015 and finished in October 2015. Baseline data collection commenced in September 2015 and the study closed with final time point data collection in February 2016.

Prior to implementation, one of the study sites had a change of management and high staff turnover, resulting in withdrawal from the study. As a result, only two settings participated in the post-implementation phase.

### Participants

Pre-implementation, we conducted qualitative focus groups and interviews with six family members and 20 professionals comprising care home staff (n = 15), GPs (n = 3) and DNs (n = 2) (S1 Table). Four family members who expressed interest and shared their contact details did not participate due to time commitments (n = 1), non-contactable (n = 1), did not arrive (n = 2). All care home staff who were approached and available participated. Four GPs were approached, one declined citing workload reasons. Three DNs were approached and expressed interest but one was unavailable due to work commitments.

During the IPOS-Dem implementation period, we conducted three non-participant observations with three senior care home staff and one GP in one care home. Also during the IPOS-Dem implementation phase we conducted two sequential interviews with both care home managers. Towards the end of the IPOS-Dem implementation period, we conducted focus groups and qualitative interviews with seven family members and five care home staff. For family members, thirteen expressed interest and shared their details to be contacted and were therefore sent details of the study. Eleven responded after two contact attempts. One declined due to his relative’s deteriorating health. Of the ten remaining family members, six and an additional family member of one participant took part. Four expressed interest but were unavailable to attend. Care home staff working and available at the time of the focus group participated in the focus group (n = 4). One care home staff member was recruited to participate in a semi-structured interview. One GP was approached to participate in focus groups but could not be recruited due to time constraints [S1 Table].

The mean length of focus groups was 77 minutes (range: 62–92), total 7 hours and 41 minutes. The mean length of interviews was 49 minutes (range: 26–91), total 8 hours and 5 minutes. In the post-implementation phase, none of the family members had awareness of IPOS-Dem being used with their relatives. All participating care home staff had used IPOS-Dem in the study, apart from the managers who had awareness of the staff’s use of IPOS-Dem.

Baseline and final time point data were collected for 32 residents and 30 residents respectively. One resident died and another moved to a nursing home due to complex care needs. S1 Fig. shows flow diagram of recruitment, including consenting process, and reasons for attrition. Table 1 includes demographic and clinical data of participating residents. Care home staff completed utility questionnaires were returned for all 32 baseline resident assessments and all 30 resident assessments and final time point.

**Table 1:** Baseline demographic and clinical characteristics of participating residents.

Variable	Residents n = 32 (%)
**Socio-demographic details**	
**Age**	
• Mean (SD) • Median (range)	• 87.2 (8.3) • 89 (67–102)
**Sex**	
• Male • Female	• 8 (25) • 24 (75)
**Ethnicity**	
• White British, Irish or other • Black Caribbean	• 28 (88) • 4 (13)
**Clinical details**	
**Formal diagnosis of dementia**	
• Yes • No	• 25 (78) • 7 (22)
**Dementia subtype**	
• Alzheimer’s disease • Vascular dementia • Alzheimer’s disease–mixed type • Unspecified dementia • Missing • Not applicable	• 4 (13) • 6 (19) • 7 (22) • 1 (3) • 9 (28) • 7 (22)
**FAST dementia stage**	
• 4–5: Mild dementia to moderate dementia • 6a- 6e: Moderately severe dementia • 7a-7f: Severe dementia	• 4 (13) • 24 (75) • 3 (9)
**Agitation**	
Mean (SD, range) Cohen-Mansfield Agitation Inventory (29–203)¶	50.3 (14.0, 29–93)
**Functional status**	
Mean (SD, range) Barthel Index (scores 0–100)†	52.7 (24.7, 0–90)
**Morbidities (excluding dementia) [9]: Resident number of morbidities**	
Mean (SD) per resident	5.1 (2.1)
**Morbidities (excluding dementia) [9]: Number of morbidities by group for all residents**	
• Cardiovascular • Musculoskeletal • Sensory diagnoses or impairments • Psychiatric • Diabetes • Cancer • All other	• 51 • 21 • 18 • 15 • 10 • 8 • 40
**Medication: Resident number of medications $**	
• Mean per resident (SD) • Missing	• 6.3 (2.6) • 5
**Medication types: Number of medications by type for all residents**	
• Cardiovascular • Analgesia • Laxatives • Antidepressant/ mood stabiliser • Antipsychotic • Dementia medication • Other	• 65 • 10 • 18 • 9 • 4 • 6 • 58

SD: standard deviation

¶ Higher scores indicate increased agitation

† Higher scores indicate greater independence

$ Medication missing for 5 residents

### Key findings

**Fig 2:**
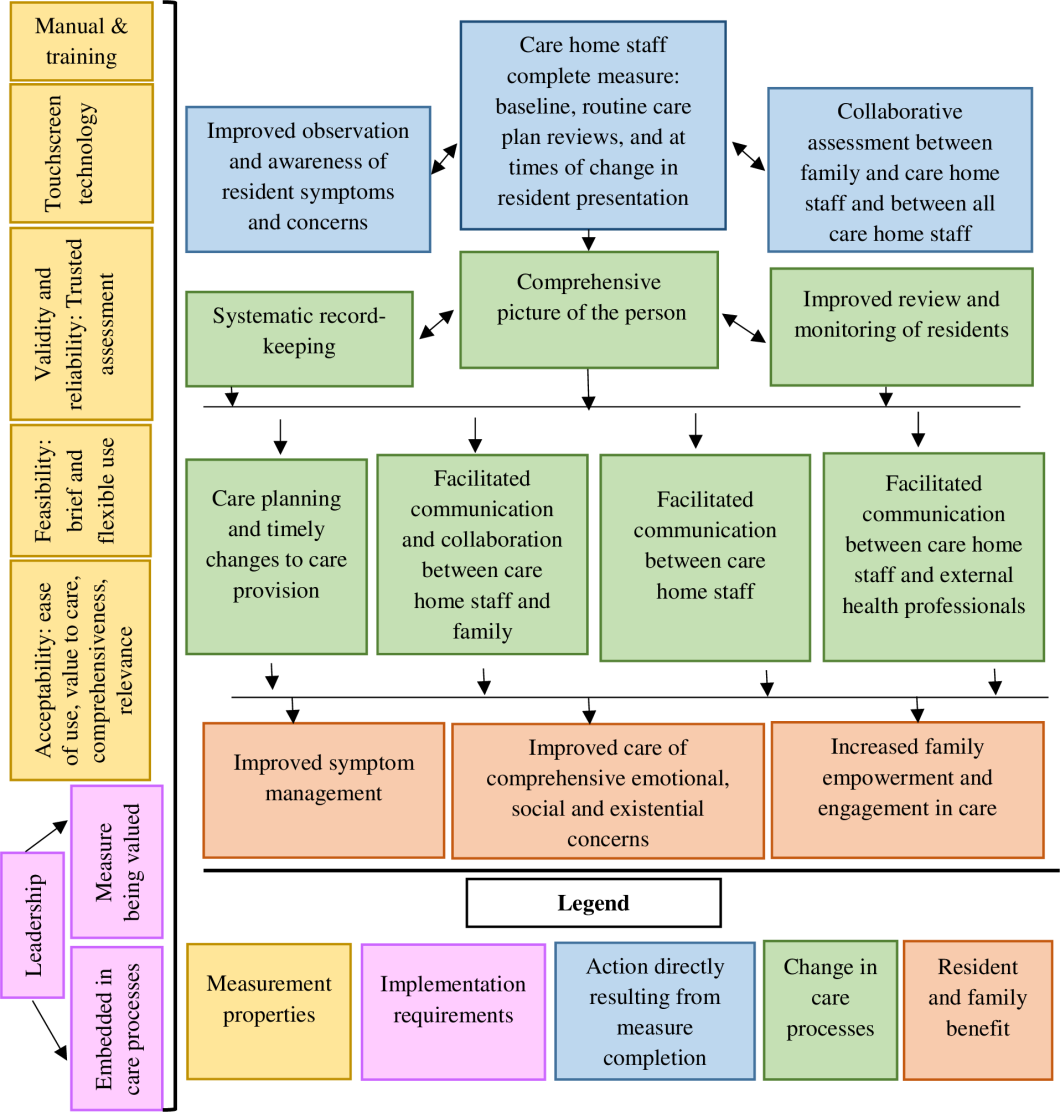
Theoretical model of IPOS-Dem mechanisms of action, potential benefit, measurement properties and implementation requirements for use in routine care of people with dementia in care homes.

Key findings from all the data are summarised here, and explored in more detail in the subsections that follow. Challenges to symptom identification and communication were identified. Key mechanisms of action of using IPOS-Dem were: (1) improved observation and awareness; (2) collaborative assessment; (3) comprehensive ‘picture of the person’; (4) systematic record-keeping; (5) improved monitoring and review; (6) care planning and changes to care provision; and (7) facilitated communication. Potential resident and family benefit were identified as: (1) improved symptom management; (2) comprehensive care needs being addressed; and (3) increased family empowerment and engagement in care. Measurement properties included: (1) acceptability of IPOS-Dem: easy to use with low missing scores and providing value to care, and relevant and comprehensive; (2) feasibility: perceived to be quick to complete, with flexible frequency. Important measurement properties were identified as: (1) ‘trusted’ as an assessment i.e. known validity and reliability and established as a recognised measure; (2) administered using touch-screen technology. Leadership was essential to ensure that the measure is integrated in care home processes, and that it is valued and recognised as a tool to improve care processes and outcomes. These are findings are combined in Fig 2 to illustrate the refined theoretical model of mechanisms of action, measurement properties, and implementation requirements. S2 Table includes model components with underpinning participant narratives.

### Mechanisms of action

#### Improved observation and awareness of resident symptoms and concerns

In both phases, all participants reported that IPOS-Dem supported improved awareness of residents’ symptoms and concerns. Family participants expressed concern that IPOS-Dem may be less useful for experienced staff, and therefore less value for the time spent completing it, but would be a good training tool for new care home staff.

Care home staff identified that it prompted them to think more about resident symptoms and concerns:

‘We understood the questions but I think you have to think deeply about what may be the answer you might think of. As a resident, as an individual maybe you don’t quite think quite so deeply until someone asks you that question’ (Care home staff B3011)

#### Collaborative assessment between family and care home staff and between all care home staff

Care home staff identified that sometimes they had gaps in their knowledge of residents, particularly regarding to residents’ earlier lives. Findings from both family and professional participants suggested that using IPOS-Dem could address this through facilitating consultation with family in the assessment of residents:

‘Isn’t this where we come in? As much as we can obviously give some history as to how our parents or whoever it is, um what their personality was, you know, and so we can contribute and say that prior to the diagnosis of dementia they were a difficult person anyway’ (Family B1009)

Furthermore, completing IPOS-Dem prompted care home staff to discuss residents amongst themselves further improving assessment and awareness of resident concerns:

‘Particularly if someone’s sitting near to somebody else when they’re completing it and they might just say ‘what do you think about this?’ so it’s actually prompting conversation which maybe in some senses you’re saying the document is meant to be really clear but as a care manager, I think it’s brilliant that anything that prompts conversation between staff about a resident’ (Manager B3001.1)

#### Comprehensive ‘picture of the person’

Pre-implementation, family and professional participants expressed concerns that IPOS-Dem would not provide sufficiently detailed assessment, and that a much more comprehensive and thorough assessment could be obtained through written text in care plans. As a result, participants reported that time would be wasted by care home staff completing IPOS-Dem without the provision of any meaningful information. Conversely, post-implementation, IPOS-Dem was seen as comprehensive and enhancing the assessment process. Participants valued how IPOS-Dem provided a comprehensive overall ‘picture of the person’:

‘Erm it sort of put you in the mind of, although we’re doing care plans and we’re doing report but it gives you a picture as well you know that you, you’re seeing a picture of a person when you’re doing this so yeah it do helps (Care home staff A3003)

This provided benefit by allowing a comprehensive knowledge of any concerns about a resident from a brief look at IPOS-Dem. Participants reported this as much more favourable compared to going through lengthy case notes.

#### Systematic record-keeping

Participants in the post-implementation phase reported that IPOS-Dem would result in improved record-keeping. Completing IPOS-Dem and severity scoring, was reported as an efficient means of recording residents’ symptoms and concerns over time, and easier to access compared to existing case notes.

#### Improved review and monitoring of residents

Participants in both phases identified the potential benefit of IPOS-Dem in monitoring residents over time. IPOS-Dem used regularly could facilitate early detection of symptoms and problems, ‘refresh the brain’ by enabling care home staff to review how residents have been over time, provide information on patterns of behaviour, and inform end-of-life care through knowledge of residents over time:

‘or that would help them towards the end of life though because all that information you’ve got about that person could be used … yeah because if they suffer from depression or they’re particularly low or there’s different things you know about that person when it comes to the end of life you’d have more of an understanding about whether they’re in pain or… (Care home staff B3011).

#### Care planning and timely changes to care provision

Participants reported that IPOS-Dem could be used to inform care plans, and result in changes to care provision:

‘And indeed [care home staff] using it to be an additional either reinforcement or even a step beyond, um and then to get into especially towards the end of life, and it does mean anticipating, it does mean early identification and action…’ (GP B1004)

Participants also shared how IPOS-Dem could also result in improved access to health care through improved identification of symptoms, and improved communication. A few participants identified the potential benefit of having an IPOS-Dem action plan, ensuring that any identified concerns trigger a change in care. However, some participants identified that there may be a risk of identifying problems but not acting upon them:

‘You know if we are because we’re in the situation where we’re thinking everybody’s exactly the same and then suddenly the data comes back saying actually you aren’t identifying that there have been quite significant changes which are written down but nobody’s doing anything about. Because the problem with care plans is you write things down but you don’t necessarily act on them’ (Manager B3001.1)

#### Facilitated communication between family and care home staff

All participants identified challenges of communication between family and care home staff. Many family members identified problems with communication with care home staff, with challenges in accessing information in a timely manner. This was exacerbated by shift work. Information, when provided, was rushed and not adequately discussed:

‘… I’m sure there is a record on my mum but even when I, you know I meet with them they don’t get it out and look at it you know, erm and I have said before now I’d like to come in and talk to you about her but they just say oh she’s taking all her tablets and …’ (Family A3002)

Care home staff identified challenges in communicating with families. Barriers to communication were lack of confidence, concerns about causing distress to family, concerns about not giving accurate information, or not knowing the answers to family questions:

‘One has to be very um polite see, in a case like that it’s crucial and one has to be very aware of the kind of information (Care home staff B3012)…information you give (Care home staff B3014) … how you give it and what you’re going to say, some are sensitive so it’s a very difficult (Care home staff B3012)…especially if someone’s at the end of life’ (Care home staff B3011) (Care home staff focus group)

A tension was identified regarding whose role it is to communicate. Family members tended to prefer communication with managers. Care home staff referred family to managers for any more complex information, while one manager in particular, considered it important that care home staff are enabled and skilled to communicate with families:

‘One of the things that really frustrates me is when staff say well speak to the manager–no you’re looking after the person, you tell them, and with this tool [IPOS-Dem] you can’ (Manager B3001.2)

Family participants welcomed the potential opportunity of accessing information and overwhelmingly reported the wish for IPOS-Dem assessments to be shared with them, and the potential benefit to care as a result:

‘If the staff were completing this on a weekly basis, can I come down and say, can I see what [they’ve] said about my mum? (Family A1006) … So would that be useful? (CES) …Oh God, yes (Family A1006) …Yeah (Family A1007)’ (Family focus group)

While the majority of care home staff perceived IPOS-Dem as a means of easily conveying information to families. Post-implementation, participants discussed the possibility of family members disagreeing with care home staff assessment, and the potential negative impact of this. This was contrasted with perceived potential benefit as a result of improved dialogue. However, for this to occur, the importance of a culture of transparency and lack of defensiveness was highlighted by both family and care home staff participants:

‘I think you know I think anyone who’s looking after your parents have to be, have to be engaged, very engaged with you and have to be very honest with you and you have to be very honest with them really’ (Family A3004).

Participants, particularly care home staff, expressed concern that sharing IPOS-Dem with family may result in family distress if the information was sensitive or unexpected. However, there was also recognition of the importance of transparent communication with family members. Family overwhelmingly identified the benefit of being able to access IPOS-Dem assessments, and did not identify any concerns about potential distress of seeing assessments.

#### Facilitated communication between care home staff

Participants post-implementation identified how IPOS-Dem could support communication between care home staff. IPOS-Dem was considered potentially useful for care home staff returning to work after time off as a means of quickly getting an update on resident changes or concerns:

**‘**… and certainly one of the things I’ve considered is if somebody’s been on annual leave saying to them make sure you read the [IPOS-Dem]. I haven’t done it yet, but almost like on your first day back, the first thing you’ve got to, the thing you’ve got to achieve before the end of your first day is read–is just checking on every resident and you can’t do that with the normal care plans but I think you could do it with this’ (Manager B3001.2)

Also post-implementation, participants identified the usefulness of IPOS-Dem in supporting communication between junior and senior care home staff:

‘I’ve had more staff come to me regarding 2 residents having difficulty swallowing in the last 2 months than I think I’ve had in the last 2 years all of a sudden erm I don’t think she’s swallowing properly, I think she’s holding it in her mouth…’ (Manager B3001.1)

Senior care home staff valued the ability to return from leave and quickly and easily get a resident update through looking at IPOS-Dem assessments. Senior care home staff and managers also identified how IPOS-Dem could support supervision. Participants suggested that IPOS-Dem could help them monitor the quality of junior care home staff assessment, and ensure that care home staff are acting upon any symptoms or problems identified:

‘supervising [junior care staff] to see that they are actually knowing the clients that they’re looking after’ (Care home staff A3003)

One manager, however, expressed concern over the potential additional burden of supervising whether care home staff had accurately assessed residents and acted upon identified concerns.

#### Facilitated communication between care home staff and external health professionals

All participants in both phases shared communication challenges between care home staff and external health professionals. Barriers to communication were shift work, high health care staff turnover, time limitations, differing expectations, and lack of shared documentation.

Participants in both phases identified how IPOS-Dem had potential to support communication with health and social care professionals. This was particularly the case in working with mental health professionals. However, as was evidenced by observations of GP consultations and expressed by participants in both phases, there was uncertainty as to whether GPs would have time to read documents.

### Potential benefit to residents and family

#### Improved symptom management

Participants reported that symptoms identified through IPOS-Dem prompted treatment:

‘Yeah I mean I can certainly think of one resident who erm has recently started to suffer with constipation and that was highlighted in this and now they’re on a laxative which you know so I know this was used for that so–‘ (Manager B3001.2)

#### Improved care of comprehensive emotional, social and care concerns

In both study phases, care home staff participants identified the usefulness of a comprehensive measure to detect resident concerns, and improve the care provided to residents:

‘Because if you’re finding things out from this and you’re making the lives of clients better, whether it be in a health way, or whether it be mentally, physically, in whatever way, then that’s a good thing, something you might not have picked up on without this’ (Manager C1005)

#### Increased family empowerment and engagement in care

In the post-implementation phase some but not all, participants identified how IPOS-Dem could help increase family member empowerment to advocate for the resident and help to engage in their care:

‘Erm yeah I do because if maybe she had diarrhoea for—then I could possibly pick up on it you know and say to them have you done anything about this?’ (Family A3002)

The same family member, however, reported that even using the measure would not help her overcome the challenges of communication.

### Measurement properties

#### Acceptability: ‘Ease of use’ and ‘value to care’

Care home staff overwhelmingly reported that IPOS-Dem is easy to use and understand. IPOS-Dem was reported to provide value to care. The value was enhanced by care home staff understanding the purpose IPOS-Dem and valuing how it can help and contribute to improving and supporting care provision. Care home staff participants reported that all care home staff should use IPOS-Dem with residents and that it is sufficiently accessible for all care home staff providing care no matter what their seniority is:

‘I should think so because I mean we’re all doing the same job, we all write care plans …’ (Care home staff B3013)

One participant, a manager, expressed concerns about all care home staff using IPOS-Dem, due to time constraints and literacy skills:

‘I think mostly it’s time because they are so involved in the practical needs of the residents you know, um plus a lot of them don’t have very good you know handwriting [literacy] skills and that kind of thing, they weren’t employed for their handwriting skills’ (Manager A3001.1)

Care home staff participants discussed the challenges of assessing verbally compromised residents, and that there is always a degree of uncertainty in the assessment of these residents:

‘I think sometimes when you’re talking about pain or you’re talking about the way that person might be feeling you have an idea of the way they might feel but you don’t really know how they are feeling because they can’t express themselves so …’ (Care home staff B3011)

However, they spoke about the benefits of knowing residents well to inform assessment, and the requirement to closely observe behaviour to inform assessment. A few items were considered potentially problematic. The usefulness and challenge of the item, ‘family information’ was discussed by participants. Family participants expressed the importance of this item, but concern that care home staff would not be able to accurately respond to this:

‘They’re making a guess, that everybody in the family has had enough information as they want whether they’ve verbalised it or not; so they can only say have the family asked for more information and has it been provided, not that they want more’ (Family B1008)

This was corroborated by care home staff participants:

‘How do you know that families get enough information relayed to them as they should have? (Care home staff B3011)

Quantitative data supported these findings. IPOS-Dem assessments were completed in full with no missing items for 21 out of 32 (65.6%) residents at baseline (n = 32). Levels of missing items for each IPOS-Dem assessment improved over time to 25 out of 30 (83.3%) complete assessments with no missing items at final time point (n = 30). Across all 28 items for 32 residents (896 items across all cases), IPOS-Dem had 19 (2.1%) missing items at baseline and this decreased to 9 (1.1%) for 30 residents (840 items across all cases) at the final time point. At baseline 14 out of the 28 IPOS-Dem items (50.0%) had no missing scores and this had increased to 21 out of 28 items (75.0%) at final time point (Table 2).

**Table 2:** IPOS-Dem scores at baseline and final time point.

	Baseline					Final time point at 12 weeks				
IPOS-Dem item	N	Missing N (%)	Cannot assess N (%)	Mean (SD)	Range	N	Missing N (%)	Cannot assess N (%)	Mean (SD)	Range
**Physical symptoms**										
Pain	29	0 (0.0)	3 (9.4)	0.62 (0.82)	0–2	29	1 (3.3)	0 (0.0)	0.86 (0.92)	0–3
Shortness of Breath	31	1 (3.1)	0 (0.0)	0.23 (0.62)	0–2	29	1 (3.3)	0 (0.0)	0.17 (0.38)	0–1
Weakness or lack of energy	31	0 (0.0)	1 (3.1)	0.97 (1.08)	0–3	30	0 (0.0)	0 (0.0)	0.87 (1.0)	0–3
Nausea	30	0 (0.0)	2 (6.3)	0.13 (0.57)	0–3	30	0 (0.0)	0 (0.0)	0.33 (0.84)	0–3
Vomiting	31	1 (3.1)	0 (0.0)	0.13 (0.72)	0–4	30	0 (0.0)	0 (0.0)	0.20 (0.76)	0–3
Poor appetite	32	0 (0.0)	0 (0.0)	0.63 (0.75)	0–2	30	0 (0.0)	0 (0.0)	0.63 (0.85)	0–3
Constipation	30	0 (0.0)	2 (6.3)	0.37 (0.56)	0–2	30	0 (0.0)	0 (0.0)	0.30 (0.65)	0–3
Dental Problems	31	0 (0.0)	1 (3.1)	0.10 (0.30)	0–2	30	0 (0.0)	0 (0.0)	0.13 (0.43)	0–2
Sore or dry mouth	32	0 (0.0)	0 (0.0)	0.09 (0.39)	0–2	30	0 (0.0)	0 (0.0)	0.00 (0.00)	0–0
Drowsiness	32	0 (0.0)	0 (0.0)	0.84 (0.88)	0–3	30	0 (0.0)	0 (0.0)	0.70 (0.92)	0–3
Poor mobility	32	0 (0.0)	0 (0.0)	1.19 (1.36)	0–4	30	0 (0.0)	0 (0.0)	0.93 (1.26)	0–3
Swallowing problems	32	0 (0.0)	0 (0.0)	0.22 (0.71)	0–3	30	0 (0.0)	0 (0.0)	0.07 (0.25)	0–1
Skin breakdown	32	0 (0.0)	0 (0.0)	0.41 (0.71)	0–2	30	0 (0.0)	0 (0.0)	0.37(0.77)	0–3
Diarrhoea	32	0 (0.0)	0 (0.0)	0.13 (0.55)	0–3	30	0 (0.0)	0 (0.0)	0.13 (0.43)	0–2
Physical symptoms sub-score: no imputation (14 items)	25	2 (0.5)$	9 (2.0)$	5.72 (4.92)	0–18	28	2 (0.5)$	0 (0.0)$	5.96 (4.99)	0–19
	22*			5.32 (4.38)*		22*			5.27 (4.36)*	
Physical symptoms sub-score: imputation 1†	32	n/a	n/a	6.04 (4.63)	0–18	32	n/a	n/a	5.70 (4.76)	0–19
Physical symptoms sub-score: imputation 2‡	32	n/a	n/a	6.12 (4.74)	0–18	30	n/a	n/a	5.67 (4.94)	0–19
	30*			5.69 (4.35)*						
**Emotional, social and existential concerns**										
Difficulty communicating	32	0 (0.0)	0 (0.0)	1.09 (1.23)	0–4	30	0 (0.0)	0 (0.0)	0.97 (1.00)	0–3
Sleeping problems	31	1 (3.1)	0 (0.0)	0.13 (0.50)	0–2	28	0 (0.0)	2 (6.7)	0.07 (0.26)	0–1
Hallucinations and delusions	32	0 (0.0)	0 (0.0)	0.69 (1.06)	0–4	29	0 (0.0)	2 (6.7)	0.59 (0.91)	0–3
Agitation	32	0 (0.0)	0 (0.0)	1.50 (1.27)	0–4	30	0 (0.0)	0 (0.0)	0.77 (0.97)	0–3
Wandering	32	0 (0.0)	0 (0.0)	0.41 (0.62)	0–2	30	0 (0.0)	0 (0.0)	0.47 (0.78)	0–3
Anxious or worried	32	0 (0.0)	0 (0.0)	1.06 (1.19)	0–4	29	0 (0.0)	1 (3.3)	0.86 (0.79)	0–2
Depressed	30	1 (3.1)	1 (3.1)	0.80 (0.96)	0–4	30	0 (0.0)	0 (0.0)	0.53 (0.73)	0–2
Lost interest	31	0 (0.0)	1 (3.1)	1.39 (1.23)	0–4	30	0 (0.0)	0 (0.0)	1.03 (1.03)	0–3
Peace	31	0 (0.0)	1 (3.1)	1.00 (0.97)	0–4	30	0 (0.0)	0 (0.0)	0.83 (0.79)	0–3
Positive interaction	32	0 (0.0)	0 (0.0)	0.94 (1.16)	0–4	30	0 (0.0)	0 (0.0)	1.30 (0.79)	0–3
Enjoyment of activities	32	0 (0.0)	0 (0.0)	1.53 (1.34)	0–4	30	0 (0.0)	0 (0.0)	1.20 (1.06)	0–4
Practical problems	31	1 (3.1)	0 (0.0)	0.23 (0.43)	0–1	30	0 (0.0)	0 (0.0)	0.17 (0.59)	0–3
ESE concerns mean sub-score: no imputation (12 items)	26	3 (0.8)$	3 (0.8)$	11.08 (7.91)	0–29	27	0 (0.0)$	5 (1.4)$	8.52 (5.91)	0–25
	21*			9.71 (6.90)*		21*			8.80 (6.47)*	
ESE concerns mean sub-score: imputation 1†	32	n/a	n/a	10.76 (7.17)	0–29	32	n/a	n/a	8.79 (5.48)	0–25
ESE concerns mean sub-score: imputation 2‡	32	n/a	n/a	10.77 (7.18)	0–29	30	n/a	n/a	8.86 (5.71)	0–25
	30*			9.66 (5.86)*						
**Family concerns**										
Family anxious or worried	31	0 (0.0)	1 (3.1)	0.45 (0.89)	0–4	29	0 (0.0)	1 (3.3)	0.45 (0.74)	0–2
Family information	31	0 (0.0)	1 (3.1)	0.13 (0.34)	0–1	29	0 (0.0)	1 (3.3)	0.07 (0.26)	0–1
Family concerns sub-score: no imputation (2 items)	30	0 (0.0)$	2 (3.1) $	0.60 (1.04)	0–4	29	0 (0.0)$	2 (3.3)$	0.52 (0.87)	0–3
	27*			0.59 (1.05)*		27*			0.48 (0.85)*	
Family concerns sub-score: imputation 1†	32	n/a	n/a	0.58 (1.01)	0–4	32	n/a	n/a	0.52 (0.83)	0–3
Family concerns sub-score: imputation 2‡	32	n/a	n/a	0.56 (1.01)	0–4	29	n/a	n/a	0.52 (0.87)	0–3
	29*			0.55 (1.02)*						
**Total scores**										
Total score: no imputation (28 items)	21	5 (0.6)	14 (1.6)	18.14 (12.15)	0–44	25	2 (0.2)	7 (0.8)	14.56 (9.71)	0–42
	17*			15.47 (10.51)*		17*			15.82 (10.94)*	
Total score: imputation 1†	32	n/a	n/a	17.38 (10.39)	0–44	32	n/a	n/a	15.01 (9.05)	0–42
Total score: imputation 2‡	32	n/a	n/a	17.46 (10.46)	0–44	30	n/a	n/a	15.05 (9.44)	0–42
	30*			15.89 (8.70)*						

* n = analysis for complete pairs only

$ Denominator is number of items in sub or total score multiplied by number of resident assessments

**†** Imputation 1: mean IPOS-Dem item score imputed

‡ Imputation 2: case mean item (sub)-score imputed

Missing data was either missing as it was rated as ‘Cannot assess’ by the care home staff, or items were not completed (i.e. reason unknown). At baseline 14 out of 896 (1.6%) items were rated ‘cannot assess’ and 5 out of 896 (0.6%) were missing with reasons unknown. ‘Pain’ had the highest number rated ‘cannot assess’ (n = 3, 9.4%), followed by ‘nausea’ (n = 2, 6.3%) and ‘constipation’ (n = 2, 6.3%). At the final time point, 7 out of 840 (0.8%) were rated ‘Cannot assess’ and 2 out of 840 (0.2%) were missing with reasons unknown. ‘Sleeping problems’ and ‘hallucinations and delusions’ had the highest number rated ‘Cannot assess’ (n = 2, 6.7%). We tested the null hypotheses that there is no relationship between missing IPOS-Dem items and dementia stage, agitation, function or care home; and were unable to reject any of the null hypotheses. It is likely that the missing data is related to some other unexplored factor such as a feature of care home staff.

Thirty out of 30 (100%) of care home staff who responded at baseline (two missing responses) reported that the time spent completing IPOS-Dem had been worthwhile compared to 20 out of 30 (66.7%) at final time point (no missing responses). At baseline 30 out of 31 (96.8) reported no challenges to completing IPOS-Dem (one missing response) and final time point 26 out of 28 (92.9%) reported no challenges to completing IPOS-Dem (two missing responses). The following two reasons for challenges were provided (one care home staff did not provide a reason): *‘Sometimes difficult to know if resident in pain due to low moods’*, *‘Don’t know about family*, *don’t know how to assess hallucination’*. At baseline 8 out of 31 (25.8%) (one missing response) of the care home staff reported that completing IPOS-Dem would result in changes to care. At final time point this had increased to 18 out of 30 (60.0%) (no missing responses).

#### Acceptability: Comprehensiveness and relevance

Participants reported that the items of IPOS-Dem are relevant, comprehensive and important. Family participants in particular welcomed the comprehensive nature of the assessment:

‘…so I think you know just the over the past week where you’ve got I think it’s question 3 to–yes it’s question 3 onwards you know about their, how they are, how they’re feeling, and interacting with other people and staff and I think that’s really really important. Lots of people do interact and I feel my mum doesn’t, and my mum’s always been a party girl you know she was always get up and go,…’ (Family A3002)

And that it addressed important concerns, including their own:

‘Anxious, has any of her [family been], anxious or worried about the person, I think that’s important really, I mean I think, [sigh] (Family A3003)

There was some discussion amongst family members about the acceptability of the term ‘palliative care’ with most, but not all participants finding the term acceptable and appropriate.

**Fig 3:**
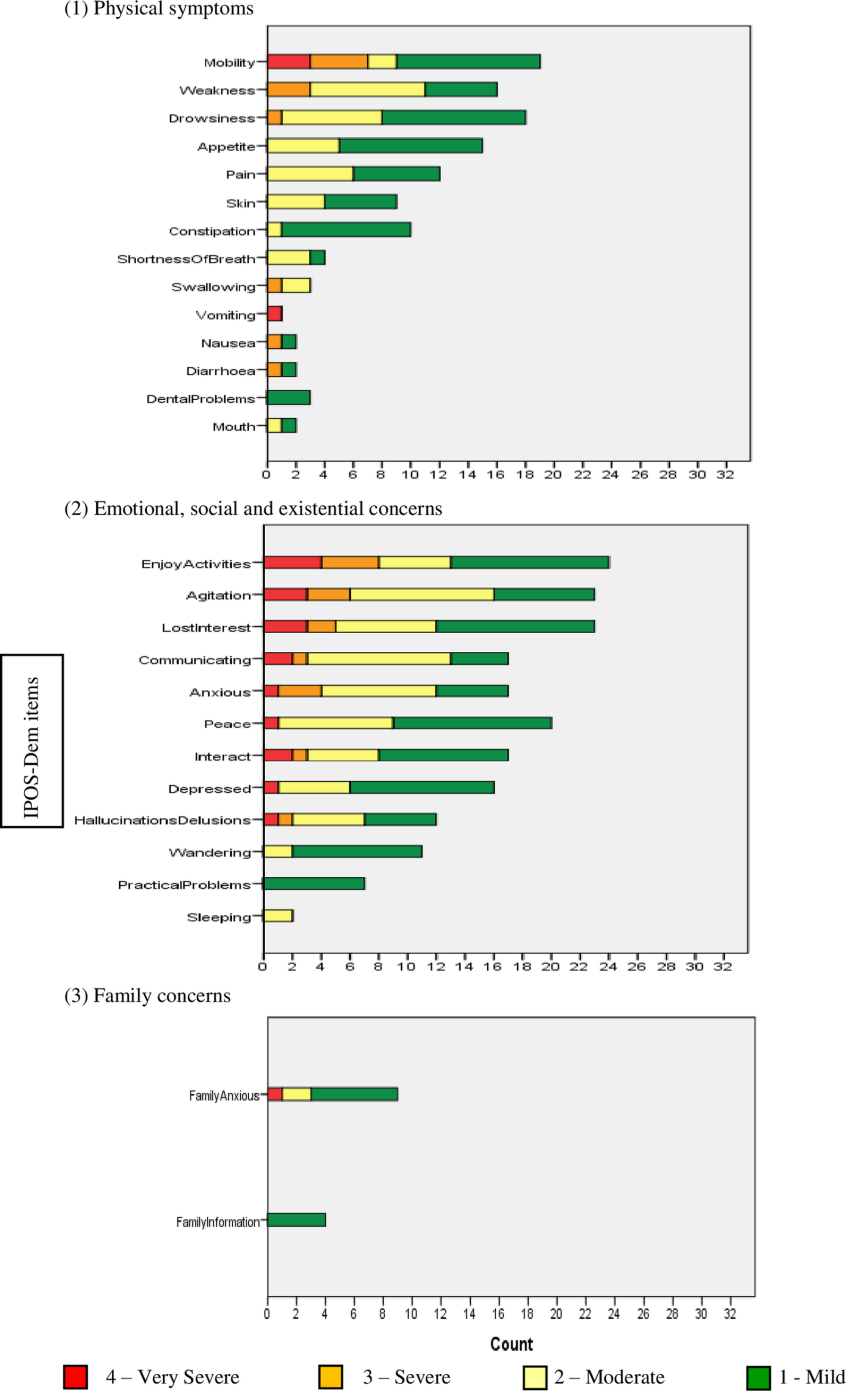
Prevalence and severity of (1) physical symptoms, (2) ESE concerns, and (3) family concerns (n = 32).

Fig 3 shows prevalence and severity of symptoms and concerns experienced by residents at baseline, by symptom and/or concern subgroups comprising: (1) physical symptoms, (2) emotional, social and existential (ESE) concerns, and (3) family concerns. ESE concerns were the most prevalent and severe with a mean item score of 0.90 (SD 0.60) compared to physical symptoms with a mean item score of 0.44 (SD 0.44) and family concerns with a mean score of 0.28 (SD 0.51). The full range of IPOS-Dem (0–4) scores for the majority of items were not used (Fig 3, Table 2). On question one (free text item), seven cases had three main problems reported, five cases had two main problems and eight cases had one main problem. The majority of these were classified as ‘agitation’ (n = 11), followed by ‘poor mobility’ (n = 4), ‘anxious’ (n = 4), ‘wandering’ (n = 4) and ‘pain’ (n = 1), all of which are items included in IPOS-Dem. The remainder could not be classified into IPOS-Dem items as they were specific to the individual reflecting the diverse concerns experienced by this population and the requirement for free text to capture these (S3 Table).

There were no significant differences between baseline and final time points for mean total scores and the three symptom/concerns subgroups with or without imputation. Areas of physical symptoms and ESE concerns appeared to remain stable across the time points with moderate correlation between baseline and final time points with all imputations (rho = 0.58–0.80). Family concerns were not correlated (rho = 0.25–0.29) between baseline and final time points.

#### Feasibility: Brief and flexible use

All participants reported the usefulness of completing IPOS-Dem at baseline. In both phases, the majority of participants identified that IPOS-Dem should be used routinely when reviewing care plans and at times of change in resident presentation (e.g. changes in behaviour, unstable or deteriorating physical health), with a minority of participants stating that IPOS-Dem should only be used at baseline and at times of change to identify potential symptoms or problems that could be contributing to a changed presentation.

The majority of participants described its feasibility for use in routine care, as quick to complete, and that it became quicker to use over time. With few exceptions, professional participants reported that IPOS-Dem was best done monthly to inform care plans, but with flexibility and more frequently if required:

‘For some people it might vary, some people you might need to do it every day (Care home staff C1007) … yeah (Manager C1005) …whereas some people you might do it once a month, while some you have to do it weekly (Care home staff C1007)

One manager considered the potential benefit and usefulness of IPOS-Dem being used on a weekly basis. Conversely, family participants expressed greater concern regarding the feasibility of IPOS-Dem and the potential time burden on care home staff and risk of taking from their caring role. The mean time it took to complete IPOS-Dem at baseline was 8.48 minutes (SD 4.98) and at final time point was 5.60 minutes (SD 1.45).

#### Validity and reliability; ‘trusted assessment’

Participants in both phases identified the potential risk of inaccurate assessment, either as a result of poor assessment or the measurement properties of IPOS-Dem (i.e. reliability):

‘… but to me poor appetite might be not eating for a week, for somebody else it is a different pers- they’ll say poor appetite is when you haven’t eaten for a couple of hours… (Family B1007)

As such, participants discussed whether IPOS-Dem would provide them with useful and trustworthy information, and the requirement for health professionals to trust the assessment of care home staff. Participants identified that a measure that is recognised across agencies and trusted would evidence their assessment and support communication with visiting health professionals. This would also address the challenge of integrated working:

‘and they’ve got the time to do it honestly, truthfully, then yes because anyone that needs to look at this whether it be GP, ambulance, consultant, relative, they know exactly what is going on’ (Family B3006)

#### Touchscreen technology

For this study, a paper-based version of IPOS-Dem was used. The topic guides did not include questions of technology in the use of IPOS-Dem. Nonetheless, the use of touch-screen technology was identified by both family and professional participants as improving acceptability through improved ease of use, improved ease of monitoring, support in identifying areas of concerns and triggering action plans:

‘Erm, it’s a long way off but somebody’s got to start planning it, and this would fit perfectly with that touchscreen situation, and if it was done in such a way that you could identify one particular aspect as well and just get the swallowing for the last 6 months or the skin integrity for the last 6 months it would be brilliant’ (Manager B3001.1)

### Implementation requirements

Managers and care home staff considered leadership as essential in implementing IPOS-Dem to facilitate integration into routine care processes through e.g. supervision, care planning. Leadership was seen as required to support adoption by all care home staff, ensuring that care home staff remember to use the measure, and ensuring they understand its purpose; thus ensuring that the measure is recognised as a valued tool to support care provision despite additional time burden:

‘and I know it’s more work, but even if it’s only a little bit, it’s still more work regardless of a little or a lot but I think things like this which, I don’t mean this selfishly, doesn’t just look after the clients, it promotes us, it promotes the care we’re giving, it promotes the way in which we work, so you know, I don’t think it shouldn’t be done. I think it’s something that all homes should do’ (Manager C1005)

Care home staff identified challenges of remembering and getting into the routine of using IPOS-Dem on a monthly basis. Quantitative data supported this with the number of IPOS-Dem measures being completed during the implementation period between baseline and final time point, increasing from 0/32 (0.0%) in the first month to 7/30 (23.3%) in the final month. Care home staff also identified the practical challenges of making IPOS-Dem accessible for family and external health professionals.

Care home staff participants overwhelmingly reported that IPOS-Dem was easy to use and did not feel that training was required. Senior care home staff and managers, however, stated that online training or DVD training would support implementation of the measure, and that this would be important to understand the potential benefit of IPOS-Dem and how it may support care.

## Discussion

We found that it is possible to introduce a measure into the routine care of residents and that this may change care processes to improve resident and family outcomes. We identified likely mechanisms of action within the care home context taking into account multi-agency working between family, care home staff and health professionals. Important measurement properties both to facilitate its use and support mechanisms of action were identified; as well as the requirements for implementation. Based on our findings, we refined our theoretical model (Fig 2).

Our findings supported many of our expected mechanisms of action detailed in the initial theoretical model, including improved observation and awareness, improved care planning and care provision, and facilitated communication and collaboration between all agencies; corroborating results of previous studies in clinical [18, 59, 60] and care home [61, 62] settings. We identified additional unexpected mechanisms of action, informing our final theoretical model and reflecting the more complex care processes in care homes. The use of IPOS-Dem facilitated a comprehensive assessment of resident symptoms and concerns, which fostered a ‘picture of the person’. This ‘picture of the person’ was valued as a means of recording complex assessments of residents in a succinct and easily accessible format, which in turn supported systematic records-keeping, monitoring, and improved knowledge of residents over time. Furthermore, the ‘picture of the person’ facilitated communication within care home settings, supporting a previous finding [61]. This included incorporating IPOS-Dem into supervision to monitor assessments and ensure that symptoms and concerns are acted upon. We identified significant challenges to communication between family and care home staff. These resulted from care home staff confidence and skill, shift work and differing expectations of roles. IPOS-Dem was identified as a potentially useful tool to overcome some of these barriers, thus improving and empowering family engagement in care provision; important to family members [63]. In addition, the measure if trusted and recognised by both parties, could facilitate communication to external health professionals. This is important as there are known challenges to integrated working between social and health care sectors [16] with barriers including a lack of trust between care homes and health care providers, and care home staff perceived lack of respect for their knowledge and skills [45, 64].

Participants reported that the measure was easy to use and provided value to care. Low missing data support this finding. However, qualitative and quantitative data suggest that there were challenges to assessing people with compromised verbal communication. All staff rated the time spent completing IPOS-Dem as worthwhile at baseline, which decreased at the final time point. The reason for this is unknown. However, it is possible that care home staff found the baseline assessment most useful, and that information subsequently obtained from IPOS-Dem was of less value as symptoms and concerns had already been identified. Conversely, care home staff ratings of whether using IPOS-Dem would change care processes increased from baseline to final time point. Again, while the reasons for this are not known, it is possible that over the course of the study, care home staff increasingly recognised the role of using IPOS-Dem to support care. We examined the use of IPOS-Dem without any support from the research team so as to understand its use and implementation without any additional resources, frequently unavailable. IPOS-Dem completion rates were low but continued to increase throughout the course of the implementation. Qualitative data suggest that care home staff required time to get into the routine of using and remembering to use IPOS-Dem, and put structures in place to support its use. This explains the low but increasing use of IPOS-Dem over the 12 week implementation period, and suggests that, with the right training resources, it might be possible to implement IPOS-Dem without the use of ‘high facilitation’ [44, 65]. Nonetheless, managers reported the potential benefit of staff training on how IPOS-Dem may support care. Provision of training on how IPOS-Dem may be integrated into care processes may also supports its implementation and use in routine care. Finally, staff may benefit from integration with primary and community health care services to support facilitation and shared organisational processes in care delivery. This type of integrated approach between care homes and health care services could facilitate use of IPOS-Dem into routine care and organisational processes, how to respond to symptoms and concerns, and support management and treatment of symptoms and concerns [16, 66].

Our participants identified the importance of the measure being comprehensive. This reflects the multiple symptoms and concerns that this population may experience due to dementia, multi-morbidities and side-effects of treatments [9]. Family members welcomed that their own needs were considered in the assessment, corroborating the importance of family concerns [67]. Touchscreen technology, while not essential, was identified as a potential key facilitator in completing IPOS-Dem, storing records, monitoring over time and communication including online access for family members. This technology is becoming increasingly common in using measures in routine care [17] and may support implementation particularly if it facilitates measure completion, storing, retrieving and analysis of scores [68]. However, it is not yet widely used in UK care homes.

We found that leadership engagement at all phases was essential in implementing IPOS-Dem, corroborating existing evidence [68, 69]. Our findings support results of previous reviews of implementation interventions [16, 70], that interventions that take into consideration time pressures and facilitate conversations between care home staff and health professionals, and those that utilise structured resources and/or tools, are more likely to be implemented and effective in improving outcomes.

In order to triangulate our data, we examined differences and similarities between family and professional participants. Family and professional participants identified similar mechanisms of action; and problems with communication although they expressed different perspectives and concerns. One surprising finding was the discrepancy between participant groups regarding the feasibility of using IPOS-Dem. Few professional participants expressed concern regarding feasibility with some considering the potential usefulness of using IPOS-Dem as frequently as weekly. Family participants, however, worried that care home staff may not have the motivation or time to use IPOS-Dem, and the potential for it detracting from their caring responsibilities.

Our study also gained an understanding of potential risk of harm of using a measure in routine care. No risks of causing harm to residents or families were identified. However, participants did identify risks of inaccurate assessment either due to poor assessment or lack of measurement reliability, corroborating a finding of a previous study [61]. Another potential risk was that even if care home staff identified symptom or concerns, they may not act, or may struggle to obtain health services required [16]. Factors that mitigated these risks were good leadership, use of the measure in supervision, and implementation involving professionals external to the care home. Furthermore, collaborative working with family may empower family members to challenge assessment and act upon identified concerns.

IPOS-Dem addresses an important gap in the comprehensive assessment of people with dementia living in care homes. In the United States, the Minimum Data Set Palliative Care [71] and Minimum Data Set for Nursing Homes [72, 73] have been used in the care home setting. These provide a means of providing comprehensive clinical assessment and supports record-keeping. However, as far as we are aware, they have not been evaluated as a complex interventions to improve care processes and outcomes for resident and family members. The Minimal Documentation system for Palliative Care (MIDOS) has been evaluated in such a way in three nursing homes in Germany [61]. The MIDOS is a brief comprehensive symptom assessment tool for self-assessment or for use by carers. However, it was not developed for dementia and contains no dementia-specific items.

Our findings indicate that further work is warranted. There are challenges to assessing people with compromised verbal communication. Training to support assessment particularly of the more challenging symptoms such as pain and hallucinations would improve acceptability. Support may include formal training, or the use of ‘add-on’ established and valid symptom assessment measures e.g. pain to support assessment [36]. Our findings also suggest that a training component should incorporate information on the measure being ‘trusted’, how it can provide value to care, and how to support its integration into care processes. Established validity and reliability is important and further psychometric testing is warranted. Finally, although we have developed a theoretical model detailing expected mechanisms of action and potential benefit to residents and families, this remains theoretical and needs to be tested further. A feasibility trial is required to test the proposed processes and likely mechanisms of action, and to inform the research methods for a full trial to evaluate the effectiveness of IPOS-Dem, including selecting the most suitable outcome measures to demonstrate benefit for residents and family members [19].

As with all studies, there are a number of strengths and limitations. Rather than just hypothesise the mechanisms of action pre-implementation, we examined the use of the measure during implementation into routine care, taking into consideration the care home context and implementation requirements. We incorporated family perspectives and compared and contrasted these to professionals thus gaining a much more detailed and insightful view into some of the contextual challenges and potential mechanisms of action. To triangulate our data, we used a number of different qualitative and quantitative data collection methods [56]. The limitations of the study are that the study settings are likely to be ‘good’ care homes and more receptive to implementing new initiatives. Also, during the course of the study, the researchers developed a working relationship with the care home staff. The care home staff knew the researchers had developed IPOS-Dem and had contributed to its development. This may have affected their willingness to criticise the tool, although our findings indicate areas they identified for improvement, for example, support in assessment verbally compromised individuals. Our sample size of residents was small and there were challenges to identifying and recruiting family members. Family members also had limited awareness of the use of IPOS-Dem with their relatives during the implementation phase. This may limit their ability to evaluate specifically the IPOS-Dem mechanisms of action, potential benefit or potential challenges, rather they report on priorities for care, care processes and outcomes, and how IPOS-Dem may address these. The implementation phase of the study was 12 weeks. This, limits the understanding on sustaining implementation and integration into care processes, in routine care.

## Conclusion

In a population with dementia and complex care needs, characterised by multi-morbidity and high symptom burden, and with challenges in assessment and integrated working; IPOS-Dem introduced into routine care is feasible and acceptable, and can support comprehensive and assessment and management of symptoms and concerns. The refined theoretical model conceptualises the likely mechanisms of action of how the measure may change care processes and potentially benefit residents and families, and the implementation requirements. Further psychometric testing and a full trial of effectiveness are indicated.

## Supporting information

S1 File Fictional case vignettes. (DOCX)

S2 File IPOS-Dem utility questionnaire. (DOCX)

S1 Table Qualitative participants’ demographic data. (DOCX)

S2 Table Model components with underpinning participant quotations. (DOCX)

S3 Table IPOS-Dem question one main problems. (DOCX)

S1 Fig Flow diagram of resident participation. (DOC)
