# Supplementary material for: How can a measure improve assessment and management of symptoms and concerns for people with dementia in care homes? A mixed-methods feasibility and process evaluation of IPOS-Dem
Source: PLoS One. 2018 Jul 11;13(7):e0200240. doi: 10.1371/journal.pone.0200240 (PMC6040756; doi:10.1371/journal.pone.0200240)
Supplement: S1 File — (DOCX) [file pone.0200240.s001.docx]

**S1 file: Fictional case vignettes**

*Vignette one*

Mrs Hazel Matthews and her husband, Mr James Matthews:

Hazel is a 72 year old lady with moderate Alzheimer’s disease. She has been living in the residential care home for nine months and moved there when her husband had a stroke and was no longer able to look after her. Mr Matthews has made a good recovery and visits Hazel regularly. He remains frail however, and is no longer to be his wife’s full-time carer. Their children are very supportive but all live abroad and are only able to visit infrequently.

Functionally, Hazel needs support for most of personal care needs. She appears to be physically well and has no other illnesses apart from high blood pressure and osteoarthritis. She walks independently and fairly safely although needs some help with transfers at times. Hazel has some communication difficulties due to her dementia and has been observed to become frustrated when she can’t find the right word.

Since moving into the care home, Hazel has generally settled well. She engages in all her personal care needs although requires some encouragement to eat her meals. She is, however, reluctant to take part in any of the activities in the care home and prefers to sit by herself frequently isolating herself in her own bedroom.

Occasionally, Hazel has been observed to be tearful but no-one has been able to ascertain from her why she is distressed. At these times, her communication tends to worsen causing her to become visibly more distressed. Later, when asked, Mrs Matthews does not appear to have any recollection of crying and seems surprised that she might have been.

Hazel is visibly more cheerful when her husband visits her. When asked why his wife may become tearful, he is unable to give an answer. Mr Matthews has explained that while his wife has always been a little reserved and private, she has been quite easy-going and not easily distressed. He wonders whether she may be in pain and has requested that her GP visit her.

*Vignette two*

Mr and Mrs Matthews three months later:

During the past three months and Mrs Hazel has been reasonable stable. She continues to have tearful episodes and requires a little coaxing to eat meals and engage. Nonetheless, most of the staff report that they have no concerns regarding providing care. She has had no apparent changes in her health.

James, however, has suddenly become quite unwell and had a two-week hospital admission. He is now much frailer and not able to visit frequently. Without close family nearby, he finds it increasingly difficult to get to the care home.

Since then, Hazel has become markedly more withdrawn. She isolates herself frequently and, for the first time, has started to resist some of the care offered and provided. Worryingly, while she has always been a small eater and required some encouragement to eat her meals, she now seems to have lost her appetite entirely. She is losing weight and staff are worried that she may be becoming under-nourished and at risk of dehydration.

At times Hazel becomes quite agitated, wandering around the care home. At these times she is very distressed but has problems explaining herself. As a result, she becomes more distressed and agitated.
