# Supplementary material for: How can a measure improve assessment and management of symptoms and concerns for people with dementia in care homes? A mixed-methods feasibility and process evaluation of IPOS-Dem
Source: PLoS One. 2018 Jul 11;13(7):e0200240. doi: 10.1371/journal.pone.0200240 (PMC6040756; doi:10.1371/journal.pone.0200240)
Supplement: S2 File — (DOCX) [file pone.0200240.s002.docx]

**S2 File: IPOS-Dem utility questionnaire**

Thank you for using IPOS-Dem today. In order for us to understand whether or not using IPOS-Dem is helpful and/ or practical when providing care, we would be grateful if you could answer the following questions. There are no right or wrong answers. The information you provide will help us know more about IPOS-Dem. We would be grateful if you complete this questionnaire each time you use IPOS-Dem.

You do not need to provide your name or any details about yourself. The research team collecting and analysing this information will not be able to identify who you are. If you have any questions or concerns about answering these questions, please feel free to contact [xxx] on the contact details below.

Please state approximately how long it has taken you to complete this questionnaire:

................................................ minutes

Will completing IPOS-Dem result in any action or changes to care, please circle:

YES / NO

Was the information obtained by completing IPOS-Dem worth the time spent, please circle

YES / NO

Did you encounter any challenges or problems using IPOS-Dem?

YES/ NO, please state ..............................................................................................................................

............................................................................................................................................................................................................................................................................................................................................................................................................................................................................................................................................................................................................................................................................

*Thank you*
