# Supplementary material for: How can a measure improve assessment and management of symptoms and concerns for people with dementia in care homes? A mixed-methods feasibility and process evaluation of IPOS-Dem
Source: PLoS One. 2018 Jul 11;13(7):e0200240. doi: 10.1371/journal.pone.0200240 (PMC6040756; doi:10.1371/journal.pone.0200240)
Supplement: S1 Table — (DOCX) [file pone.0200240.s003.docx]

## S1 Table: Qualitative participants’ demographic data

|  | | Pre-implementation | | | Post-implementation | | |
| --- | --- | --- | --- | --- | --- | --- | --- |
| Number of family participants | | N=6 | | | N=7 | | |
| Data collection | Method of data collection | Number of method types | | Number of participants | Number of method types | | Number of participants |
|  | Focus group  Interview | 1  2 | | 3  3 | 1  2 | | 5  2 |
| Age | Mean (SD)  Median (range) | 60.5 (6.1)  61.0 (53-68) | | | 63.7† (7.4†)  62.5† (57-78†) | | |
| Sex | Male  Female | 1  5 | | | 2  5 | | |
| Relationship to resident | Daughter  Son  Sister  Son-in-law  Friend | 4  1  n/a  n/a  1 | | | 4  1  1  1  n/a | | |
| Care home | Care home A  Care home B  Care home C | 2  3  1 | | | 2  5  n/a | | |
| Number of professional participants | | N=20 | | | N=11 | | |
| Data collection | Method of data collection | Number of method types | | Number of participants | Number of method types | | Number of participants |
|  | Focus group  Interview  Manager interview  Observation | 3  1  n/a  n/a | | 18  2  n/a  n/a | 1  1  4  3 | | 4  1  2  4 |
| Sex | Male  Female | 2  18 | | | 2  9 | | |
| Profession | Role/ profession | Type of data collection | Number of participants | | Type of data collection | Number of participants | |
|  | Manager  Team leader  Senior carer  Care assistant  Activities coordinator  GP  District nurse | Focus group  Interview  Interview  Focus group  Focus group  Focus group  Focus group  Focus group | 2  1  1  2  7  2  3    2 | | Focus group  Interview  Interview  Observation  Focus group  Observation  Focus group  Focus group  Focus group  Observation  Focus group  Observation | n/a  2  1  1  0  2  4  0  0  1  0  0 | |
| Ethnicity | White British  White Irish  Indian  Black Caribbean  Black African  Black British  Missing | 8  1  1  4  5  1  0 | | | 4  0  0  3  1  0  3 | | |
| Years of experience | Mean (SD)  Median (Range) | 10.3 (8.8)  7.0 (1-29) | | | 18.2 (10.7)*  19.0 (1.5-30)* | | |
| Care home | Care home A  Care home B  Care home C | 7  6  7 | | | 2  9  n/a | | |

* Missing data for three participants

† Missing data for one participant
