# Supplementary material for: How can a measure improve assessment and management of symptoms and concerns for people with dementia in care homes? A mixed-methods feasibility and process evaluation of IPOS-Dem
Source: PLoS One. 2018 Jul 11;13(7):e0200240. doi: 10.1371/journal.pone.0200240 (PMC6040756; doi:10.1371/journal.pone.0200240)
Supplement: S2 Table — (DOCX) [file pone.0200240.s004.docx]

S2 Table: Model components with underpinning participant quotations

|  | **Family** | **Professional** |
| --- | --- | --- |
| **Action directly resulting from measure completion** | | |
| **Improved observation and awareness of symptoms and concerns** | **Pre-implementation** | |
|  | *‘that would be quite difficult for me because if I visit once a week, I don’t know whether she’s been in pain’ (Family B1009)* | *‘It might help to exclude other things, like you’d go through it and try to find out whether she’s breathless, whether she’s sick or vomiting, sort of a symptom checker list really?’ (GP A1002)*  *‘I think it’s interesting because in my head I’m thinking, well these are the thing we would do, you know if somebody became distressed, the first thing we’d say is are you in any pain? Are you… you know, so in a sense there it’s a good way of, of making you think about the questions to ask, you as opposed to, um just assuming you’ve asked all the questions’ (Manager B1005)* |
|  | **Post-implementation** | |
|  | *‘and I think this is a really good learning tool, this is what we’re looking for, this is the sort of thing that will affect people erm and some people will already know this, they will have sort of walked in maybe having already had erm, erm experience with dementia and they will know this; but most people need it breaking down and so I think the fact that it’s broken down into this level of detail must be really useful when you’re starting to work in the care environment but I, I imagine that some of the people who are, who have worked there for many years probably could recount this in their sleep, erm and to actually have to constantly reiterate it would be diffi – more difficult … it’s still time when you could be interacting with residents’ (Family A3004)*  *‘It makes you aware of things you, you know there’s so much to do in your job that when you’re focused on something you think actually she isn’t, you now she isn’t eating very well, she does leave her dinner every day whereas you know you’re in such a rush to give out the dinners, collect them in and, and move them back into their seats they may not…’ (Family A3002)*  *‘…but I don’t know how much training they have in order to become a care worker and so it doesn’t become part of you really so maybe, you know and I think ongoing training and prompts like a questionnaire that just make you think’ (Family A3002)* | *‘We understood the questions but I think you have to think deeply about what may be the answer you might think of. As a resident, as an individual maybe you don’t quite think quite so deeply until someone asks you that question’ (Care home staff B3011)*  *‘…or if it’s not any changes for the, the client you are dealing with that would be easy but if it’s a change that the person has um change then you have to be careful the way you are filling with not just to tick and tick and – (Care home staff B3014)*  *‘I think maybe we don’t, you don’t always think really a lot about people being at peace do you until the question’s until you’re asked that question’ (Care home staff B3011)* |
| **Collaborative assessment between family and care home staff and between all care home staff** | **Pre-implementation** | |
|  | *‘Isn’t this where we come in? As much as we can obviously give some history as to how our parents or whoever it is, um what their personality was, you know, and so we can contribute and say that prior to the diagnosis of dementia they were a difficult person anyway’ (Family B1009)* |  |
|  | **Post-implementation** | |
|  |  | *‘Particularly if someone’s sitting near to somebody else when they’re completing it and they might just say ‘what do you think about this?’ so it’s actually prompting conversation which maybe in some senses you’re saying the document is meant to be really clear but as a care manager, I think it’s brilliant that anything that prompts conversation between staff about a resident’ (Manager B3001.1)*  *‘I think sometimes it helps to know more about their past which you don’t always know about, know what they’ve done within their life you know because sometimes that can, be a good thing to know you know sometimes when someone’s passed away you’ve suddenly found out more about them when they’ve passed away than when they were here’ (Care home staff B3011)* |
| **Change in care processes** | | |
| **Comprehensive ‘picture of the person’** | **Pre-implementation** | |
|  | *‘Has [resident] been feeling anxious or worried? She does all the time, sometimes when this happens, sometimes when that happens. You can gauge a lot more from that than from one tick box’ (Family A1006)* | *‘It doesn’t actually tell us anything does it? Staff go on the care plan and they can, for staff going to the care plan they can read actually what has happened’ (Manager A1009)* |
|  | **Post-implementation** | |
|  | *‘Well I mean it does look um nicely detailed, it looks like um I, what I imagine, um in terms of my own smaller capacity as carer, I um obviously do notice things about my mum and ask her questions and try and facilitate um information from her about how she’s feeling um but to actually have it detailed in this way with particular criteria must be very useful I would think for the care workers, um and for me as well as I’m looking through it, it seems very sensible and very relevant so yes I think it’s, I think it is a useful tool just to concentrate the mind really (Family A3004)*  *‘Erm but I think if, if they had something like this where they you know, where the question is about informing, I think that would be helpful’ (Family A3002)*  *‘Yeah I think that’s really important um yes I mean I think all of those things are really important because I think definitely if there was anything, if any of these um came out negatively I know that my mum would be quite unwell and unhappy (Family A3004)* | *‘Erm it sort of put you in the mind of, although we’re doing care plans and we’re doing report but it gives you a picture as well you know that you, you’re seeing a picture of a person when you’re doing this so yeah it do helps (Care home staff A3003)*  *‘Okay, but nobody ever sits down and thinks, well I say nobody ever, that’s not true because they do but it would be so easy to say oh they just suffer with a sore mouth. What’s the link between oh they’re suffering with a sore mouth but actually they’ve got quite a lot of pain and they’ve been vomiting, have they got something that’s linking those 2 together, you know (Manager B3001.1)* |
| **Systematic record-keeping** | **Pre-implementation** | |
|  |  | *‘I think you should do it regardless of the change, because then it’s all documented isn’t it? Because you want to have the documentation of the good changes as well as the bad changes’ (Manager C1005)* |
|  | **Post-implementation** | |
|  |  | *‘Okay so say for instance, let’s go with the skin broken down. For weeks now Mrs So and So’s skin broken down, you’ve got a variance, you’ve got it’s changed, “not at all” to “moderately”. The moment you have something that maybe goes over 2 boxes, page so and so this is what we did … almost like yeah so you’re evidencing you’ve seen the change and you’ve actioned something’ (Manager B3001.2).* |
| **Improved review and monitoring of symptoms and concerns** | **Post-implementation** | |
|  | *‘Well I think um I, I’m looking at some of the um the problems that are identified, I imagine that a lot of these things happen occasionally anyway, I mean everybody probably does have sort of sleep problems and diarrhoea and vomiting or whatever from time to time because you know we’re human, but I suppose the important thing is, is to make sure that it’s monitored and that that monitoring, that the number of times things happen might, might erm alert people to when there is a real problem…’ (Family A3004)* | *‘or that would help them towards the end of life though because all that information you’ve got about that person could be used … yeah because if they suffer from depression or they’re particularly low or there’s different things you know about that person when it comes to the end of life you’d have more of an understanding about whether they’re in pain or… (Care home staff B3011).*  *‘Well no in the sense that having copies whether we would have access or having copies that is what I’m asking because at times these are important when you are just you know to refresh the brain about what you’ve just you know something (Care home staff B3012)*  *‘I think what is interesting is it is making staff think because this constant question over the last week, over the last week and it’s making staff think about a time span rather than in the moment’ (Manager B3001.1)* |
| **Care planning and timely changes to care provision** | **Pre-implementation** | |
|  |  | *‘And indeed [care staff] using it to be an additional either reinforcement or even a step beyond, um and then to get into especially towards the end of life, and it does mean anticipating, it does mean early identification and action…’ (GP B1004)*  *‘… but it’s not enough to say that that person’s in pain. The minute you know that you have a responsibility and I suppose this is exactly the same. Is this person constipated? Yes. How often? Frequently. What are you going to do about it?’ (Manager B1005)*  *‘Yeah I mean I think it could help the care plans because I think, because everything’s got to coincide doesn’t it, so there’s no point the care plan saying that you know someone’s fine, but this is saying that they’re not’ (Manager C1005)* |
|  | **Post-implementation** | |
|  | *‘that’s right so some way of dragging together the bits that are more worrying than other bits, putting them together into a co – what do you call them and email in sort of data terms but you know putting the data together and creating an answer which says, you know there’s a red light on this, you know giving red, orange and green lights really you know that patient or that care, or that care patient has got red lights we’ve got to keep an eye on him or her, she’s green she’s fine, she’s okay at the moment, that’s really the …’ (Family B3008)*  *‘I think, I think they yeah they would be more aware that is something they should be doing’ (Family A3002)* | *‘You know if we are because we’re in the situation where we’re thinking everybody’s exactly the same and then suddenly the data comes back saying actually you aren’t identifying that there have been quite significant changes which are written down but nobody’s doing anything about. Because the problem with care plans is you write things down but you don’t necessarily act on them’ (Manager B3001.1)*  *‘and as I said again to me um a method like this helps um for us to go through and see if these people needed more help from outside agents more than you know so um so yeah so that would be a part of we supervising to see what staff are putting in and see if we needed more help from outside’ (Care home staff A3003)*  *‘Um, just what I’ve said you know their changes would be identified and um we could act upon it’ (Manager A3001.1)*  *‘I think this would help us write the care plan much better’ (Care home staff B3013)*  *‘mhmm, staff have expressed that it’s quite easy to use, which is good and that it doesn’t take them too long erm because they’re not having to actually write sentences, so they’ve found it useful in as much as they’ve done this and then they’ve gone to the longer care plan, using this so, for instance if it’s, if somebody doesn’t normally have any pain and on this they’ve put that they’ve, you know they’ve got slight pain, it’s given them the thing to say what is going on that’s changed so it, it’s sitting with the longer care plans but it’s prompting the longer care plans to rather than just say no change, no change, everything’s the same, it’s now making them think about the individual questions so that’s been quite helpful’ (Manager B3001.2)*  *‘well if we think people are getting very high scores, especially in pains and erm stuff we’d seek help from the doctors that obviously this person needs to be medicated for painkillers or you know um if it’s anything to do with their mental stages deteriorating then it help us to get in touch more with the erm mental health team so…’ (Care home staff A3003)*  *‘Yeah I do think so, I think looking back at these tools can you know if staff do it because sometimes they might not report straight away, I know they do report but if you go, if we go though and look and think oh but we missed this, this person could get more help, then yeah we could look at it and seek help from, from just what we’re looking at I mean you do have staff that will discuss with their one another and they might not come in and say to us but if we look here and there’s, you know they’re thinking and doing it then we can look at it and think this person’s score is quite high and sometime again some people in here not recognises dementia and if you look back at this you can see you know we should seek help for this person, something else might help them to be more settled and you know’ (Care home staff A3003)*  *‘I think you know the more I’ve thought about it today the more I’ve thought about it being a weekly tool and I do think it triggers the mind and once it triggers the mind it triggers the care’ (Manager B3001.2)*  *‘and as I said again to me um a method like this helps um for us to go through and see if these people needed more help from outside agents more than you know so um so yeah so that would be a part of we supervising to see what staff are putting in and see if we needed more help from outside’ (Care home staff A3003)*  *‘I don’t think so, not with the staff that are doing, that are doing it I think they’re fairly confident and they, they feel sort of like fairly assured as to how to do it so I don’t think that is an issue, and one of the other things that’s come up is with the care plan that they write, they’re writing about how the person is, but this always asks them to look back over the last week’ (Manager B3001.2)*  *‘It will, won’t it because you’ll have a better understanding of that person and will have thought more about that individual, you know, and about their health and about their wellbeing so if you’re more aware about that person, individual you should be able to treat them, treat them you know the way they need to be’ (Care home staff B3011)* |
| **Facilitated communication and collaboration between family and care home staff** | **Pre-implementation** | |
|  | *‘If the staff were completing this on a weekly basis, can I come down and say, can I see what [they’ve] said about my mum? (Family A1006) … So would that be useful? (CES) …Oh God, yes (Family A1006) …Yeah (Family A1007)’*  *‘I’ve also had slight problems in that when I want information it’s often not readily available’ (Family C1008)*  *‘and it has been a problem so often I’ve just, if I’ve needed something I’ll ask her a question and give them an hour to try and look for it or a day. Um again this was about nobody to ask. If you go in there quite a bit at weekends, then you’ve got weekend staff who just say I don’t know’ (Family C1008)*  *‘Yeah, yeah because it’s a two way – it should be a two way street it shouldn’t all be left to the care home people and it shouldn’t all be left to us – if we work hand in hand you’ll get along better. That’s an ideal though isn’t it? Doesn’t always work (Family A1006)* |  |
|  | **Post-implementation** | |
|  | *‘… I’m sure there is a record on my mum but even when I, you know I meet with them they don’t get it out and look at it you know, erm and I have said before now I’d like to come in and talk to you about her but they just say oh she’s taking all her tablets and …’ (Family A3002)*  *‘I think you know I think anyone who’s looking after your parents have to be, have to be engaged, very engaged with you and have to be very honest with you and you have to be very honest with them really’ (Family A3004).*  *‘Erm some time ago and it was kind of 6 weeks and she still hadn’t seen the doctor so I said I would take her, and then the doctor said that they couldn’t see me and that they would come to the home but the home had to ring them and – and eventually the doctor came but they never even fed back to me what was going on’ (Family A3002)*  *‘Communica – I can’t fault this home at all for its care because I know that he’s washed, fed, and looked after generally but erm [Manager] knows this because I’ve complained to [Manager], the communication here is terrible’ (Family B3006)*  *‘I’d have to say it doesn’t happen at all, they would probably say they do inform us but I, I just, I just come in, see my mum and just believe that everything else is a big black hole because when I ask questions I don’t get answers’ (Family A3002)*  *‘know that that’s the truth and it’s not the right thing to do and I know it isn’t and here I look and I think she gets her tablets on time, she erm is clean, and dressed well, she err has good food, they keep the place you know quite clean erm and she can’t get out so she’s safe, erm and you can’t have everything so there are lots of good things but I think communication is probably the biggest concern I have’ (Family A3002)*  *‘I have trouble on the phone as well when I’ve phoned up to see how it is, you never get, I know obviously they do shift and the shift patterns change’ (Family B3006)*  *‘but I find anything to do with record keeping is done very hastily on the hoof so you’re just about to go out the door and someone says well will you sign this?’ (Family B3007)*  *‘… but as I say it is always n, in a rush that you get pushed a piece of paper for you to sign and you really haven’t got time to look through it’ (Family B3007)*  *‘I must say I’ve, I haven’t found it like you, I do a lot of emailing and I find that if I email and ask something, I get a response reasonably quickly back, I don’t know whether you use email do you to contact [Manager]?’ (Family B3008)*  *‘Well erm yeah I mean I think that, I think that er obviously the information, all the information that um they can give me about what’s happening with my mum is really helpful, um I spend quite a lot of time with mum, she comes out here most Sundays for lunch, I take her out quite a lot walking so anything, I need to know what’s, how she is at any particular time whether I can take her out, whether she can erm whether she can come erm with me anywhere, erm and how she’s going to be so clearly I think it’s, the more information that staff can give me about her physical and mental wellbeing the more I’m able to make the time that we have together erm happy’ (Family A3003)*  *‘Erm but I think if, if they had something like this where they you know, where the question is about informing, I think that would be helpful’ (Family A3002)*  *‘see I think it should be the other way around, I don’t think that we should be thinking, are we embarrassed to ask, I think they should be saying please look at the record, please have a look at it, I think that, that the emphasis of this tool should in writing be on it, this tool should be made available on a regular basis to the family, so it should be pushed from them, not, we shouldn’t feel ohh, I don’t know whether we should ask or not, it’s not the point, it’s the other way around, the, the um, the home should be pushing the information to us and saying you’re part of this, this journey, you must be with us all the way, it’s not a matter of them providing it all and we just observing, you know we are part of the journey’ (Family B3008)* | *‘One has to be very um polite see, in a case like that it’s crucial and one has to be very aware of the kind of information (Care home staff B3012)…information you give (Care home staff B3014) … how you give it and what you’re going to say, some are sensitive so it’s a very difficult (Care home staff B3012)…especially if someone’s at the end of life’ (Care home staff B3011)*  *‘One of the things that really frustrates me is when staff say well speak to the manager – no you’re looking after the person, you tell them, and with this tool you can’ (Manager B3001.2)*  *‘but maybe with this [IPOS-Dem] they don’t have to go because some of them can’t be bothered to sit there all, going through the care plan, where this you could just say okay we’re using this now, as well as a care plan, if you don’t have time to go through, look through this and you can see why we’re saying that your dad or your mum needed more support you know so then they can look through and see what we’re going on about (Care home staff A3003)*  *‘but you can always look through and you could always say to them if you not agree with what we’ve said in here then, with all means you could see a different picture from what we see because we are looking here looking after mum or dad, you could come in and see something else that we’ve missed so yeah you know so we could always say if you think that something I should add in then let us know and we could improve from that as well’ (Care home staff A3003)*  *‘It depends on the kind of questions the relatives want to find out, is depends of the, the, the question, if it’s the question that you can refer the person to the manager you refer the person to the manager, you have to limit what you can tell the family, sometimes you are keyworker for so so so person, when you are key worker the relative walk out and ask you oh how my mum has been, sometimes they will ask oh who is so so so person who look after my mum, if you are not there or if you are there they will come and call you say how my mum has been, has she been okay? So you say oh yeah yeah yeah she has been fine but with the question sometimes is the things that if she want to find more, you refer the person to the manager’ (Care home staff B3014)*  *‘Especially end of life if you’re in that situation where somebody’s seriously ill or it’s kind of, and they’re asking you like really explicit questions about that person you’ve got to know (Care home staff B3011) … what to say (Care home staff B3013) … you’re telling the correction information because that, that could be you that’s not (Care home staff B3011)’*  *‘So you have to think seriously (Care home staff B3011) … especially if the resident um not well, and going to the hospital and the relative is there (Care home staff B3013) … mm (CES) … you are just trying to be cautious you don’t want to say something that, it might affect you later on (Care home staff B3013) … do not know (Care home staff B3014) … or you don’t know for sure (Care home staff B3013) … you might upset them by saying something (Care home staff B3011) … and then you might upset them by saying something so sometimes it’s best for them to talk and you listen and if there’s something you can say but say it in a very nice way (Care home staff B3013)’*  *‘You don’t know, you don’t have a clue, you have to go and look for team leader to tell the team leader that such and such person wants to know about – (Care home staff B3013) …we’re in a bit of an awkward situation sometimes (Care home staff B3011)’*  *‘I’ve got some relatives who will never leave the building without coming to speak to me, even though I know they’ve had a conversation with somebody else’ (Manager B3001.2)*  *‘Do you think that such a tool could support working with family members? (CES) No they, they’re not around enough really to um have an objective view on it’ (Manager A3001.1)*  *‘Erm the risk for family members is that they will misunderstand the document erm and they will think that because it’s got a tick that’s the end of the answer, do you know what I mean? So there has to be open two way conversations. If a family member sees it they’ve got to be able to ask the questions, you can’t be defensive with a document like this, you’ve got to be able to justify why you’ve written what you’ve written’ (Manager B3001.1)*  *‘Yes, yeah you, if you want to fill up and you can, ever you want the family to be there and you see this is what your mother has been going through, she has been vomiting, I know you are aware of this that’s why I’m putting it down, you can be agree, she’s agree and you agree what you are doing it’s nothing to hide from the family, have to share’ (Care home staff B3014)*  *‘No I think the thing is you’re absolutely right because some staff the moment they’re challenged see it as an attack and go into defence, whereas I, I work really hard with staff to say this is a question, they’re showing an interest in the person that you’ve been looking after, qualify your answer, tell them why you’ve done that, explain to them why you feel this, so instead of being all defensive what’s it got to do with them, it’s got everything to do with them erm and you need to be able to tell them how the money that their mum is paying for you to look after them is being spent wisely, qualify what you’ve done, you know’ (Manager B3001.1)* |
| **Facilitated communication between care home staff** | **Post-implementation** | |
|  |  | *‘I’ve had more staff come to me regarding 2 residents having difficulty swallowing in the last 2 months than I think I’ve had in the last 2 years all of a sudden erm I don’t think she’s swallowing properly, I think she’s holding it in her mouth…’ (Manager B3001.1)*  *‘supervising [junior care staff]to see that they are actually knowing the clients that they’re looking after’ (Care home staff A3003)*  *‘… and certainly one of the things I’ve considered is if somebody’s been on annual leave saying to them make sure you read the [IPOS-Dem]. I haven’t done it yet, but almost like on your first day back, the first thing you’ve got to, the thing you’ve got to achieve before the end of your first day is read – is just checking on every resident and you can’t do that with the normal care plans but I think you could do it with this’ (Manager B3001.2)*  *‘You notice that the person have erm that problem you have to report this to the senior care to the senior, then the senior will come and look, if it’s nothing that the home can deal with they have to call um…’ (Care home staff B3014) ‘GP’ (Care home staff B3013) ‘…the doctor or emergency if it’s um…’ (Care home staff B3014)*  *‘…because we have supervision with staff every six weeks so we will ask them about their residents so if we’ve got this document I noticed over the last couple of months you’ve noticed this what have you done about it? How have you identified it?’ (Manager B3001.2)*  *‘or you can leave one of your colleagues you rely on to be observing the keyworker – the resident you know to see if there is anything important or necessary to say or to note about’ (Care home staff B3012)* |
| **Facilitated communication between care home staff and external health professionals** | **Pre-implementation** | |
|  |  | *‘yeah, yeah I’d glance at it particularly, mainly to see if they noted any changes from what is usual for them, that might be helpful’ (GP A1002)*  *‘Another reason I’m saying that is that sometimes you have clients and you notice changes in them you’re not quite sure what it is and you send them to the hospital and they send them back to us saying there’s absolutely nothing wrong with them whereas if you send this they can see why you’ve thought there’s something wrong’ (Manager C1005)* |
|  | **Post-implementation** | |
|  | *‘The real issue is whether I suppose in a way maybe the other thing to think about is whether there should be some trigger so that the care workers refer forms which they think the GP should see, to the GP so that they do the sifting because the GP won’t do it for sure, I mean you give him a pile of forms like this he’ll say forget it. Um but if the carers say well there’s 2 forms here that we think you should look at because this lady’s had diarrhoea and then you know been vomiting and that kind of stuff’ (Family B3008)*  *‘Oh yeah, yeah and because if they want to say this has been happening, and they can look back on their notes and say actually you know it was noted that you know for the last 4 times we’ve filled this form in that she’s had diarrhoea and so it would be a useful tool for … and being able to look at symptoms in order for the doctor to work out what’s wrong’ (Family A3002)*  *‘but this form is exactly what a GP would want to know isn’t it, it’s exactly what a GP would ask’ (Family B3008)*  *‘Would there be any way of having a small front sheet that if there was any information that the home felt they needed, felt a doctor needed to know just put on it rather than him ploughing through it just put please see..?’ (Family B3010)* | *‘Absolutely no consistency and I don’t know if that is nationwide or whether that’s just London, but London is dreadful at the moment erm and so you can train one batch of district nurses and then next lot are going to come in in a month’s time and don’t know what it is you’re talking about so it is going to be tough, it is going to be really really tough but if it became an, a nationally recognised tool then it wouldn’t matter which [Clinical Commissioning Group] you work from this would be the thing, and it also says well this is what we’ve been assessing so we can say to the district nurse who says you know how long’s this pressure area been there? Well actually this tool was done last week and there was no pressure area last week, so before you start screaming that it’s been going for months and nobody’s done anything, you know’ (Manager B3001.1)*  *‘No I think it can be helpful as well you know for the doctor come in and you’re quite busy you can say just look through this in the meantime, I’m doing something and you’ll see what I’m trying to say about the dementia, especially if the mental health team come in and we want to give them a picture of what’s going on with this person then you could show them this and you know instead of sit there and explaining again they have that, they just go though and then they can ask you any question they want from it so’ (Care home staff A3003)*  *‘um possibly if we contacted the mental health team um because we were concerned about certain changes in a resident’s behaviour and they might say oh well monitor them for a few days and document the changes, I think this would be an ideal tool to use to do that’ (Manager A3001.2)*  *‘No they wouldn’t go to the doctor oh look doctor so and s, so and so but they might say oh [GP name] can you go and see so and so because, and it’s just information they’ve got there because they’ve seen it’ (Manager B3001.2)*  *‘Not our business, erm when you get down to hallucinations, agitation and wandering then if this is a change, if somebody, if we’ve been saying for the last 6 months that somebody’s hallucinating severely, it actually says why didn’t you get in touch with us sooner, but it also says we’ve been monitoring to make sure it wasn’t a one off, and we can evidence that they have been hallucinating, that they have, I probably wouldn’t wait 6 months but, you know but just for explanations sake. So it is a way of saying this is, this isn’t new or this is new, this person never hallucinates and all of a sudden they are hallucinating, erm so yeah and you know we in [borough name] we use the care home interim, the care home support team’ (Manager B300.1.)*  *‘Well I think um we have the booklet during this so there’s a lot of information from the booklet, um and also yeah this will help as well for the, the professionals to see and work from it as well to see you know because they could come in and you’ll miss something to tell them and if you have it all written down by the time they get here it’s already here so it’s um’ (Care home staff A3003)*  *‘I don’t think it’s worth doing on top of the care plan I mean you have sometimes you call the mental health team out and then they don’t shuffle through those things if they have something like this’ (Care home staff A3003)*  *‘I think, I don’t think they use the document to communicate in as much as I don’t think they show [GP name] the document but I think it informs so if the staff are noticing something they’ll mention it to the team leader and the team leader will then put them down to see the doctor’ (Manager B3001.2)* |
| **Resident and family benefit** | | |
| **Improved symptom management** | **Post-implementation** | |
|  |  | *‘Yeah I mean I can certainly think of one resident who erm has recently started to suffer with constipation and that was highlighted in this and now they’re on a laxative which you know so I know this was used for that so – ’ (Manager B3001.2)* |
| **Improved care of comprehensive emotional, social and existential concerns** | **Pre-implementation** | |
|  |  | *‘Because if you’re finding things out from this and you’re making the lives of clients better, whether it be in a health way, or whether it be mentally, physically, in whatever way, then that’s a good thing, something you might not have picked up on without this’ (Manager C1005)* |
|  | **Post-implementation** | |
|  |  | *‘Because it does make you stop and think and it makes you stop and ask yourself what am I doing? What am I doing, what have I done about this and I think the minute you see there’s a change and you see I’ve got to do something, that improves the life of a resident’ (Manager B3001.2)* |
| **Increased family empowerment and engagement in care** | **Post-implementation** | |
|  | *‘Erm yeah I do because if maybe she had diarrhoea for - then I could possibly pick up on it you know and say to them have you done anything about this?’ (Family A3002)*  *‘[sighs] I think some family members could, yes, I do I think as a whole family members could question it, it might make them think a bit more and I could just imagine I wouldn’t, I might not say anything because I’m that sort of person, I think I give up so, and I shouldn’t, someone said something was done in the hospital recently, when I went to the hospital this week and someone said well didn’t you say and I thought what’s the point, if they’re in that profession and they don’t see that, you know my mum was supposed to be keeping this thing on her eye and she kept moving it and she went “put it back”, and you think don’t talk to her like that, but I thought, if, if I have to tell you then you’re not going to change you know what’s the point in having an argument you know with someone and I think maybe I don’t speak up when I should at times, you know and I think if this, if they ticked that they’d communicated with me, I think there’s a chance I might not say anything (Family A3002)*  *‘I think the staff could press more for the visitors to have a look at the tool and even perhaps a comment on it because that would help them to sort of respond to anything that the staff had spotted so I think that, I think that you know the danger of this kind of form filling is that it just gets put away and everybody thinks thank God I’ve filled in a form, that’s it you know finished, but it’s no good unless it’s actually a positive err useful piece of work really isn’t it so I actually think that, that the outcome of this would be to add to it you know erm comment of visitor, you know when visited, erm and it would also show the sort of regularity of visits and the difference in sort or who visits and the rest of it which might help in terms of care generally I suppose’ (Family B3008)* | *‘Yeah it might be reassuring to them that you know we are watching because they might have noticed a change and you know it would be reassuring for them to see oh yeah they’ve realised too and they’re watching what’s going on’ (Manager A3001.2)* |
| **Measurement properties** | | |
| **Acceptability: ‘ease of use’ and ‘value to care’** | **Post-implementation** | |
|  | *‘They’re making a guess, that everybody in the family has had enough information as they want whether they’ve verbalised it or not; so they can only say have the family asked for more information and has it been provided, not that they want more’ (Family B1008)*  *‘It must be hard for staff in a care home working with people with Alzheimer’s dementia to know if those residents are in any discomfort or pain, going by when [resident of B3006 – BB3003] had his fall recently, when I got to the hospital erm to see him, he had a massive erm swelling the side of his head and erm a big bruise, black going down towards his eye, and on the back of his head he’s taken the skin off but not once, and it must have been painful, not once did he sa – did he mention that he was hurting’ (Family B3006)* | *‘I should think so because I mean we’re all doing the same job, we all write care plans …’ (Care home staff B3013)*  *‘I think mostly it’s time because they are so involved in the practical needs of the residents you know, um plus a lot of them don’t have very good you know handwriting [literacy] skills and that kind of thing, they weren’t employed for their handwriting skills’ (Manager A3001.1)*  *‘I think sometimes when you’re talking about pain or you’re talking about the way that person might be feeling you have an idea of the way they might feel but you don’t really know how they are feeling because they can’t express themselves so …’ (Care home staff B3011)*  *‘We are looking after the resident you see some who cannot express themselves but as a carer you notice that oh this resident, where he would try to um to behave you can see the face, the way he would do um the way he would make the face you know that oh this resident he have pain somewhere or he will hold, sometimes he will hold his stomach like that then you know oh maybe she got a pain in her stomach, maybe she constipated or maybe, so things like that they will make the sign that you, you feel that maybe she have pain’ (Care home staff B3014)*  *‘How do you know that families get enough information relayed to them as they should have? (Care home staff B3011)*  *‘You know how dare you say you’ve given me enough information when actually you haven’t spoken to me…?’ (Manager B3001.2)*  *‘So there is a point at which the value, the value of the document comes in the document being valued’ (Manager B3001.1)*  *‘Because they can help you to answer it, especially pains and um depression, you ask these questions and they can answer to say yes or no how I feel where with the others some of them you got to sort of you use your own skill to find out what is happening so yeah’ (Care home staff A3003)*  *‘Well especially if they are in pain you notice you know their face expression, you notice when you’re trying to do personal hygiene, if they scream, anything like that so you’ll recognise that this person’s in pain, um you know more sleepy, you can say they’re more tired looking, more withdrawn, if it’s severe or, you understand? So…’(Care home staff A3003)*  *‘But for in that case probably occasionally I think um probably she’s a bit um anxious or having some fear that she’s going to be hurt so it’s sort of anticipating, thinking about that I hurt that is going to happen’ (Care home staff B3012)*  *‘I guess what I think you have to be observing more the resident as an individual more, probably within the care plans and get more information about that will help’ (Care home staff B3012)*  *‘Yeah that’s the, one of the great benefits of staff being here long term, not flitting in and out because they actually know the residents and they would immediately spot any changes and know whether that was a concern or not’ (Manager A3001.2)*  *‘So basically but then, but if you ask it like that then you’ve got to ask then why not and is it because I haven’t seen the family, or erm I haven’t had the information to give the family if I so it’s 2 ways it could be no I haven’t informed the family but only because I haven’t seen them, or it could be no I haven’t informed the family because I don’t know what to inform them’ (Manager B3001.1)*  *‘How do you know if I’m at peace?’ (Care home staff B3012) … ‘I don’t really do I? You make an assessment you make a judgement about people but you don’t really know’ (Care home staff B3011)*  *‘This one here, can she or he enjoy activities appropriate for his or her level of interests and abilities, that’s quite a hard one don’t you think?’ (Care home staff B3011)*  *‘I think it is, I think it is and I think it is, I think that is one of the questions that is open to interpretation I really do, because for some people being at peace means they’re rest – they’re not rest, restless, but for actually for others they could be very restless but still be at peace, because some people don’t get that peace is an emotion as opposed to a s – a physical state, if, if you know where I’m going with that so it’s about helping people to understand that this is about their total wellbeing, are they at, are they at erm at the point of not necessarily acceptance, but are they embracing fully where they are? Are they fighting with where they are? Are they you know are they still saying I wish, I wish, I wish, you know I wish I could do this, I wish I could do that, because if somebody says oh I wish I could walk, they actually telling you they’re sad that they can’t and so they’re not at peace because they’ve got that sense of not being able to do something, and I think, I think that question is going to be one that is interpreted very very differently’ (Manager B3001.2)* |
| **Acceptability: comprehensiveness and relevance** | **Post-implementation** | |
|  | *‘…so I think you know just the over the past week where you’ve got I think it’s question 3 to – yes it’s question 3 onwards you know about their, how they are, how they’re feeling, and interacting with other people and staff and I think that’s really really important. Lots of people do interact and I feel my mum doesn’t, and my mum’s always been a party girl you know she was always get up and go,…’ (Family A3002)*  *‘Anxious, has any of her, anxious or worried about the person, I think that’s important really, I mean I think, [sigh] (Family A3003)*  *‘but your mum’s been in homes for 8 years so I wouldn’t see her in the same sort of category, I wouldn’t see her as having palliative care would you? (Family B3008) … but it is really, it is the end of her life, isn’t it? (Family B3007) … yeah I suppose so (Family B3008) … it’s how long they can hang on for (Family B3010) … I think it’s also understanding that people do, as we said earlier, people do die from Alzheimer’s you know it’s not just a condition (Family B3007) … oh yeah (Family B3008) … they die from it (Family B3007)’*  *‘Definitely relevant because they don’t even really remember, will remember themselves, I know my mum wouldn’t remember any of these, she wouldn’t know that she hasn’t slept very well you know and I think they’re, them looking out for symptoms so the night staff are they here, because they must walk round at night and if they hear some noise in the room then they know you know, not sleeping at night is you know, erm and question 3, yeah I, I think they are all relevant [pause], but as I said you know lots of things may go on but I’m not aware of them yeah’ (Family A3002)* | *‘There’s quite a lot, appetite, the mobility, wandering, yeah there is a lot here that would um indicate that things have changed and there’s a problem’ (Manager A3001.1)*  *‘Um no they’re all quite relevant aren’t they you know have the family been anxious yeah I think they are all relevant’ (Manager A3001.2)* |
| **Feasibility and frequency of use** | **Pre-implementation** |  |
|  |  | *‘For some people it might vary, some people you might need to do it every day (Care staff C1007) … yeah (Manager C1005) …whereas some people you might do it once a month, while some you have to do it weekly (Care staff C1007)*  *‘I think…if it’s going to be coinciding like and implementing what’s in the care plan then they all have to be done on a monthly basis and more regular if changes’ (Manager C1005)*  *‘I mean, I suppose really the question was how often and who by and how would it help? … erm… we have a monthly assessment on their care, and I suppose really I would initially think that that is something we could use upon admission, and then monthly and then increase its usage if the level of change seemed to be more significant, or a tool that we could go back to, if we you know, we’ve done it and we might have done it last week, but all of a sudden we’re noticing a significant change, for it to be a tool to say well come on, let’s see what’s going on here’ (Manager B1005)* |
|  | **Post-implementation** | |
|  | *‘I think, I mean I think what, what is always helpful if you have to do this kind of monitoring and tracking is for it to be as simplified as possible and that it can be incorporated in the work that [caregiver staff] do, … it’s very difficult I think these kinds of monitoring and tracking systems are just simply time consuming and sometimes do take away from the business of you know communicating and doing all the basic stuff’ (Family A3004)*  *‘Erm [pause] I think weekly, the staff would not want to do that, and possibly, they probably wouldn’t want to do every 2 weeks either but I see things as 6 weeks as a teacher and you know if it was every 3 weeks or 4 weeks or every month you know that would be better than them not doing it but I can’t see that happening’ (Family A3002)* | *‘Erm do I think you should tell people how to – how – I think if you don’t give them a minimum timescale there will be those who will only use it when they know you need, they’ve got to, erm the thing with monthly which is, is only really coming to me as we’re talking, the thing with monthly is it’s only capturing one week in the month, but you can’t capture a month in one tick box and I’m just thinking to be honest this would be so much easier for every staff to have one of these every Monday to fill in for the previous week or you know one day for the previous week on their key residents and then when they come to do their care plan they’ve got the information there’ (Manager B3001.2)*  *‘I think you can use it like maybe for a resident that you see not having much change maybe every month or every 2 months when we’re doing our care plan as well you just use that as well as part of our tool, um somebody that you see change more rapidly as the weeks go by I think it’s good to use more as a 2 week or you know um monthly, it just depends on how, what we notice in their behaviour and changes’ (Care home staff A3003)*  *‘Well as I said again it depends on the deterioration in some of our clients, some might deteriorate more rapidly and the ones that deteriorate more rapidly I could see I mean you could have it as a weekly but I think weekly might be a bit too much so say two weekly and the ones that is more slower erm deteriorating then that could be as our care plan because what we do we do 2 monthly for the ones that not deteriorate and as time goes as well for example if we see somebody going downhill then you use it as a weekly erm method and work with it that way so’ (Care home staff A3003)*  *‘It depends on the person, if the person has changed before erm you, you do it next month, if the person, I’m giving an example, if the person change this week you can’t wait for next month to do your book [IPOS-Dem], you have to…’ (Care home staff B3014)*  *‘But as a, you know as a one-off, um I think it could be quite a good tool, but not to be used every week’ (Manager A3001.1)*  *‘Possibly, maybe you know if we notice some changes in the resident we might refer back to this’ (Manager A3001.1)*  *‘Um, intermittently, if we notice a change in a resident then we would refer back to this, perhaps go through it again and then compare how it was and how it is now’ (Manager A3001.1)*  *‘Erm I think initially once a month or where have you is, is good, as I said earlier I think it is something that particularly if you’ve got somebody who’s going through a change in their, their condition it could be used more frequently’ (Manager B3001.1)*  *‘You know rather than let’s rewrite the care plan every time there’s a change which is the situation we’re in at the moment, if somebody has a change in their condition we have to update the care plan as the condition changes so you can be writing something in the care plan every day because somebody’s changed whereas it would be brilliant if we would say we’re noticing changes, we’re going to use the [IPOS-Dem] on a weekly basis or a daily basis to monitor these changes, that would be so much easier’ (Manager B3001.1)*  *‘Not really I mean as I said if we really put our mind to it and you do one a day really it’s, you know it’s not that we’re going to sit and do all 33 so you know if everyone really put their head to it I can see it going one a day, you know once a week somebody come in and take up this particular, especially key workers you know if one said I’ll do 2 of my clients and you know I think you know it could work really’ (Care home staff A3003)*  *‘Yeah, so yeah I mean probably in the long term I would say yes it’s hard work to do 27 of these every week but nobody’s doing 27, at the most they’re doing 4 because they only have 4 key residents so at the most they’re doing 4, so it, it’s not a huge thing. Now one of the big issues of course would be the amount of paperwork you would generate. Um but I do think when you’ve got somebody who is changing or somebody who is unwell and you’re looking to see where those changes are, it could be, it could be a useful tool to do it weekly so yeah’ (Manager B3001.2)* |
| **Validity and reliability: ‘trusted assessment’** | **Pre-implementation** | |
|  | *‘… but to me poor appetite might be not eating for a week, for somebody else it is a different pers- they’ll say poor appetite is when you haven’t eaten for a couple of hours… (Family B1007)*  *‘I’m not a fan of things like this because I don’t think they’re very helpful in the long run because you give them to people and they just think I’ve got to tick one so I’ll tick this’ (Family A1006)* |  |
|  | **Post-implementation** | |
|  | *‘and they’ve got the time to do it honestly, truthfully, then yes because anyone that needs to look at this whether it be GP, ambulance, consultant, relative, they know exactly what is going on’ (Family B3006)*  *‘no, I think that, I mean the harm could be that it’s filled in incorrectly and then there’s some sort of medical intervention which might, for instance which might not be appropriate but it seems to be as you said, a GP if they read this at all would certainly not, not act on it without err second accounting and, and getting other information from other sources before say prescribing something or whatever for depression when she’s not depressed or he’s not depressed or whatever’ (Family B3008)*  *‘I would say not that helpful for GPs. I’m not sure that they would tru – to be perfectly frank I don’t know whether they would trust that this had been filled in correctly, whether they’re talking about the same person, I don’t know, I just don’t feel they would use it (Family B3007)* | *‘Absolutely no consistency and I don’t know if that is nationwide or whether that’s just London, but London is dreadful at the moment erm and so you can train one batch of district nurses and then next lot are going to come in in a month’s time and don’t know what it is you’re talking about so it is going to be tough, it is going to be really really tough but if it became an, a nationally recognised tool then it wouldn’t matter which [Clinical Commissioning Group] you work from this would be the thing, and it also says well this is what we’ve been assessing so we can say to the district nurse who says you know how long’s this pressure area been there? Well actually this tool was done last week and there was no pressure area last week, so before you start screaming that it’s been going for months and nobody’s done anything, you know’ (Manager B3001.1)*  *‘and it’s got an NHS certificate on you know it, we had exactly the same with the integrated care plan. Once they’d been trained on the integrated care plan they were happy to use it. I, I get it they’re regulated as well and they’re petrified of doing anything outside their regulation’ (Manager B3001.1)*  *‘The risk is, and you’re always going to have this when you’ve got a tick box, let’s just tick a box … and that risk can only be overcome by making sure that somebody is reading the document to make sure that it’s reflecting the person.’ (Manager B3001.1)* |
| **Touchscreen technology** | **Post-implementation** | |
|  | *‘erm I mean you know we are in the modern world, I actually think that there’s no reason why each patient should not have a private folder which the family can access and so this is actually not just so that when I, I live a long way away as well and err I would like to know on a Tuesday what’s happening you know I mean B1009 comes in lots so she could ask to look at the documents, she wouldn’t want to access them online but you might want to access it on, you know those that use computers a lot I mean it would just be useful to, to see what’s been filled in this week so you’ve got a, a running log of what’s happening, I mean that’s not a, possibly the cheapest of options as it involves all sorts of security issues and all the rest of it but I definitely think that’s another way of communicating, namely that, and the staff could then say when, you know when they get a complaint about lack of, of a response they could actually say well it’s on the, you know people can look it up when they like, it’s online’ (Family B3008)* | *‘Erm, it’s a long way off but somebody’s got to start planting it, and this would fit perfectly with that touchscreen situation, and if it was done in such a way that you could identify one particular aspect as well and just get the swallowing for the last 6 months or the skin integrity for the last 6 months it would be brilliant’ (Manager B3001.1)*  *‘I mean I think it’s a little while before care homes are going to be all electronic, but we’re talking about a, a document with a future, not a document with a past so we have to you know think, this as it is fine, this as it is is fine but when the time comes that we’ve got touch screens in every communal area and what have you or tablets in our pockets, I mean I know some of the more affluent homes and have already got tablets in their pockets but you know it’s just a case of I’ve been in to see Mrs so and so and then it can be done far more often … and if it needs to be done every day it can be done every day. If it’s on a touchscreen it’s not actually, you know we’ve been talking in terms of once a month when their care plans are done’ (Manager B3001.1)*  *‘You know because if this could go onto a tablet and you could just bring it up, pain, last 6 months, and it gives you that picture for the pain for the last 6 months that would be really good’ (Manager B3001.1)* |
| **Implementation requirements** | | |
| **Leadership: Embedded into care processes** | **Post-implementation** | |
|  |  | *‘Erm for me, what I will do once the research if over you know if we continue with the document will be to say discuss it with them because we have supervision with staff every six weeks so we will ask them about their residents so if we’ve got this document I noticed over the last couple of months you’ve noticed this what have you done about it? How have you identified it?’ (Manager B3001.1)*  *‘I do think it is purely by erm people being aware that there’s been changes and if this highlights it I, I don’t know I think, how can I, I don’t want to say big brother but somebody has to be looking at what other people are doing. There has to be a point at which staff are aware when – it’s twofold really, staff have to know when they’ve put it down somebody’s listening to it or reading it because otherwise they’ll just tick anything if they’re thinking oh they’re not going to take any notice so there is um there is a management responsibility to make sure we follow through when the staff put the effort in to do it and I think that then will complement the staff to make sure they’ve done it properly because they know the management’s going to look at it so it’s reciprocal really … erm if I know my boss is coming to look at something I know I’m going to do it properly because my boss is coming to look at it, if I think my boss isn’t interested.’ (Manager B3001.1)*  *‘erm, just to compare you know this with the current care plan. That’s the only way you’d know if it was actually being implemented’ (Manager A3001.1)*  *‘They just need that prompt from me have you done it, erm but again that, as I said earlier that just getting into the routine (Manager B3001.1)*  *‘Yeah because I’m just not used to using it, you just have to get into that mind-set of thinking it’s got to be done at the end of every month’ (Care home staff B3011)* |
| **Leadership: Measure being valued** | **Pre-implementation** | |
|  |  | *‘and I know it’s more work, but even if it’s only a little bit, it’s still more work regardless of a little or a lot but I think things like this which, I don’t mean this selfishly, doesn’t just look after the clients, it promotes us, it promotes the care we’re giving, it promotes the way in which we work, so you know, I don’t think it shouldn’t be done. I think it’s something that all homes should do’ (Manager C1005)* |
|  | **Post-implementation** | |
|  |  | *‘I think a point of, a point of reference would be explaining to staff why it’s needed, you know what’s led to this, why have we introduced this document. That we haven’t just bought out this new document but actually that we’ve become aware that for, for you as a staff you have so many things to do where you have to write sentence after sentence and we’re, you know we’re trying to recognise that by giving you a document that basically is easier to do. I think it’s about selling it from you know why, why you’ve done it, why you’ve done it in this format, and how a resident will benefit from it’ (Manager B3001.1)*  *‘I think I’ve been a motivator, I’ve had to motivate them and encourage them um I think, my role is, is to convince them a) that they’re able to do it, and b) the value of doing it, um I think those are the 2 key issues really you know to say you can do this, and this is why you’re doing it and I think I have to believe in it, if I don’t believe in it they won’t’ (Manager B3001.2)* |
