# Supplementary material for: How can a measure improve assessment and management of symptoms and concerns for people with dementia in care homes? A mixed-methods feasibility and process evaluation of IPOS-Dem
Source: PLoS One. 2018 Jul 11;13(7):e0200240. doi: 10.1371/journal.pone.0200240 (PMC6040756; doi:10.1371/journal.pone.0200240)
Supplement: S3 Table — (DOCX) [file pone.0200240.s005.docx]

**S3 Table: IPOS-Dem question one main problems**

| **Main problem (included in IPOS-Dem)** | **Number** |
| --- | --- |
| Agitation | 11 |
| Poor mobility | 4 |
| Anxious | 4 |
| Wandering | 4 |
| Pain | 1 |
| **Main problem (not included in IPOS-Dem)** | **Number** |
| Refuses care | 2 |
| Confused | 2 |
| Sleepy and withdrawn | 1 |
| Low mood, withdrawn | 1 |
| Restless about how life has turned out to be | 1 |
| Sometimes likes a lot of attention | 1 |
| Losing his voice | 1 |
| Unable to feed herself, needs prompting | 1 |
| He will over and over asking question where he is | 1 |
| Assistance at mealtimes, monitor food and fluid intake because of confusion | 1 |
| Going into the cupboard in the kitchen | 1 |
| Does not like to drink | 1 |
| Extra close supervision with daily events | 1 |
| **Number of cases with no main problems** | **12** |
| **Number of cases with one main problem** | **8** |
| **Number of cases with two main problems** | **5** |
| **Number of cases with three main problems** | **7** |
| **Total number of main problems** | **39** |
