# Supplementary material for: How can a measure improve assessment and management of symptoms and concerns for people with dementia in care homes? A mixed-methods feasibility and process evaluation of IPOS-Dem
Source: PLoS One. 2018 Jul 11;13(7):e0200240. doi: 10.1371/journal.pone.0200240 (PMC6040756; doi:10.1371/journal.pone.0200240)
Supplement: S1 Fig — (DOC) [file pone.0200240.s006.doc]

**S1 Fig.: Flow diagram of resident participation**

Assessed for eligibility (n=58)

Excluded (n=11)

♦  Not meeting inclusion criteria (n=9 )

♦  On manager advice (n=1)

♦  Family requested resident not approached at coffee morning (n=1)

Number approached and capacity assessed (n=47)

Capacity not assessed (n=1)

♦  Resident declined participation(n=1)

Lost to follow up (n=2)

♦  Died (n= 1)

♦  Moved care home (n= 1)

(n= )

Consultee approached (n=39)

♦  Personal consultee (n=15)

♦  Professional consultee (n=24)

(n=

Recruited (n= 36)

♦  Professional consultee (n=

Baseline data collected (n= 32)

Final time point data collection (n=30)

Informed consent given (n=1)

Lacked capacity to give informed consent (n=45)

Recruited but died before baseline data collection (n= 4)

Approached but not recruited (n=3)

♦  Resident died before consultee advice obtained (n=3)

NOK identified but unable to approach due to insufficient contact details (n=4)

(n=

Not recruited on consultee advice (n=3)

♦  Advised against participation (n=2)

♦  Professional consultee advised NOK involvement, but NOK did not respond (n=1)
